# Hematologic and systemic metabolic alterations due to Mediterranean class II G6PD deficiency in mice

**DOI:** 10.1172/jci.insight.147056

**Published:** 2021-07-22

**Authors:** Angelo D’Alessandro, Heather L. Howie, Ariel M. Hay, Karolina H. Dziewulska, Benjamin C. Brown, Matthew J. Wither, Matthew Karafin, Elizabeth F. Stone, Steven L. Spitalnik, Eldad A. Hod, Richard O. Francis, Xiaoyun Fu, Tiffany Thomas, James C. Zimring

**Affiliations:** 1 University of Colorado Denver Anschutz Medical Campus, Aurora, Colorado, USA.; 2 Department of Pathology and Carter Immunology Center, School of Medicine, University of Virginia, Charlottesville, Virginia, USA.; 3 Bloodworks Northwest Research Institute, Seattle, Washington, USA.; 4 Versiti Blood Center of Wisconsin, Milwaukee, Wisconsin, USA.; 5 Department of Pathology and Cell Biology, Columbia University, New York, New York, USA.

**Keywords:** Hematology, Genetic diseases, Glucose metabolism, Mouse models

## Abstract

Deficiency of glucose-6-phosphate dehydrogenase (G6PD) is the single most common enzymopathy, present in approximately 400 million humans (approximately 5%). Its prevalence is hypothesized to be due to conferring resistance to malaria. However, G6PD deficiency also results in hemolytic sequelae from oxidant stress. Moreover, G6PD deficiency is associated with kidney disease, diabetes, pulmonary hypertension, immunological defects, and neurodegenerative diseases. To date, the only available mouse models have decreased levels of WT stable G6PD caused by promoter mutations. However, human G6PD mutations are missense mutations that result in decreased enzymatic stability. As such, this results in very low activity in red blood cells (RBCs) that cannot synthesize new protein. To generate a more accurate model, the human sequence for a severe form of G6PD deficiency, Med(-), was knocked into the murine G6PD locus. As predicted, G6PD levels were extremely low in RBCs, and deficient mice had increased hemolytic sequelae to oxidant stress. Nonerythroid organs had metabolic changes consistent with mild G6PD deficiency, consistent with what has been observed in humans. Juxtaposition of G6PD-deficient and WT mice revealed altered lipid metabolism in multiple organ systems. Together, these findings both establish a mouse model of G6PD deficiency that more accurately reflects human G6PD deficiency and advance our basic understanding of altered metabolism in this setting.

## Introduction

Glucose-6-phosphate dehydrogenase (G6PD) is the first and rate-determining enzyme in the pentose phosphate pathway (PPP), which utilizes glucose to generate NADPH; the latter is the major reducing equivalent that fuels multiple pathways by which cells handle oxidative stress. Deficiency in the activity of G6PD is the single most common enzymopathy in humans, estimated to be present in approximately 400 million people worldwide (1). The complete absence of G6PD is not compatible with life, and the vast majority of mutations leading to G6PD deficiency in humans are missense mutations leading to an unstable G6PD enzyme. Based upon the mutation and resulting G6PD levels, disease can range from mild to severe deficiency (2, 3). RBCs from humans with G6PD deficiency are particularly susceptible to oxidant stress for 2 prevailing reasons. First, because RBCs lack mitochondria, the PPP is their main source of NADPH. Second, mature RBCs are unable to synthesize new proteins. When G6PD-deficient humans encounter an illness or consume a drug or food that increases oxidant stress (e.g., antimalarial quinone drugs or fava beans), they can manifest symptoms of acute hemolysis, ranging from mild to lethal (4, 5). Recent findings have also linked G6PD deficiency to many other diseases outside the RBC compartment, including immunological (1), cardiovascular (6), endocrine (7), renal (7), neurological (8), and pulmonary pathologies (9).

Because G6PD deficiency is so prevalent in humans, and because its biology remains poorly understood, a translatable animal model of G6PD deficiency is essential. Two separate mouse models of G6PD deficiency have been described (10, 11), both derived by random mutagenesis. Importantly, both result in altered levels of G6PD expression but with a normal amino acid sequence and normal protein stability (10, 11). As such, they do not recapitulate the human situation where young RBCs have high levels of G6PD activity, which then diminish as the RBCs age. To our knowledge, no mouse model has been described with an unstable G6PD enzyme.

Class II G6PD deficiency is the most severe form that lacks ongoing nonspherocytic hemolytic anemia at baseline. The best studied class II deficiency is the “Mediterranean variant” that results from a Ser188 to Phe mutation (12, 13). Herein, we describe a potentially novel mouse in which the genomic sequence for the human “Mediterranean type” G6PD-deficient variant was knocked into the murine G6PD locus, thus recapitulating the human enzymopathy in a murine system. The phenotype of this mouse resembles that of humans with G6PD deficiency, in that the mice are healthy at baseline but demonstrate hemolysis upon challenge with oxidant stress. A detailed analysis of these mice was performed, under normal and stressed conditions, including metabolomics profiling of multiple organs. In addition to describing a potentially new model, these findings provide understanding of the metabolic consequences of G6PD deficiency in multiple organ systems, as well as insights into the biochemical mechanisms by which oxidant stress alters RBCs, both in the G6PD-normal and G6PD-deficient states.

## Results

### Generation of a potentially novel murine model of human G6PD deficiency.

**Figure 1:**
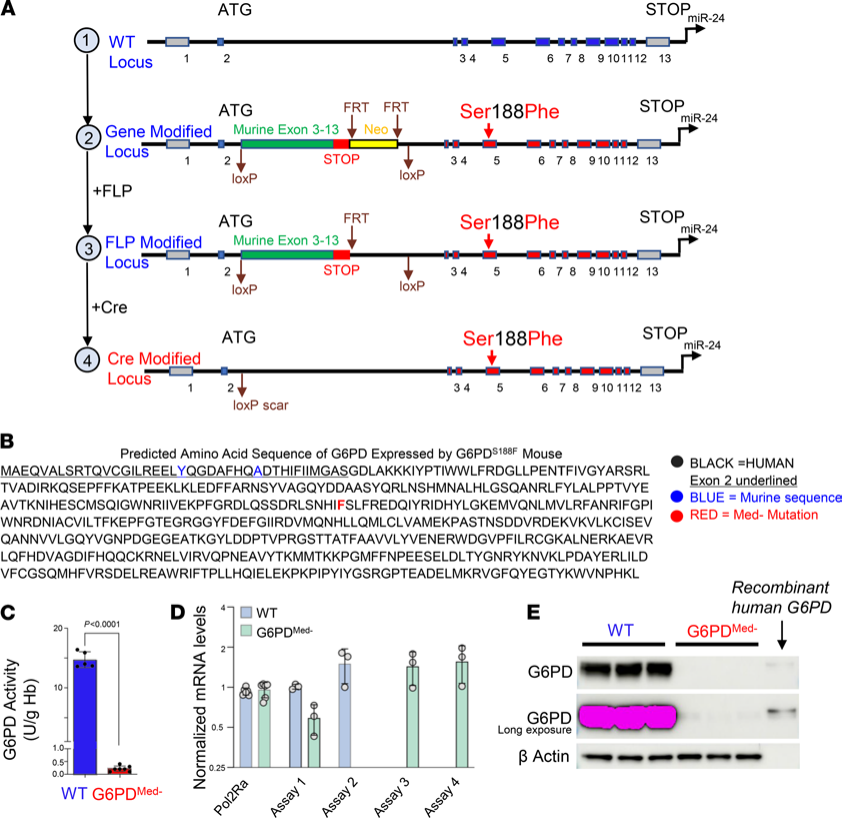
Generation of a G6PD-deficient mouse model. (**A**) Schematic representation of the WT G6PD locus (top) and related modifications (bottom). (**B**) Predicted protein sequence of knocked-in G6PD gene. (**C**) G6PD activity in RBCs from WT versus G6PD_Med-_ mice. *n* = 5–7 mice per group. Unpaired, 2-tailed *t* test. (**D**) Mouse versus human mRNA levels in G6PD_Med-_ and WT mice; assay 1 detects both WT and G6PD_Med-_ mRNA, assay 2 detects only WT, and assays 3 and 4 detect only G6PD_Med-_ mRNA (details of assay primers and probes shown in Supplemental Figure 2A). *n* = 5 for both groups for the Pol2RA experiment; *n* = 3 for WT and G6PD. Data shown as mean ± SD in **C** and **D**. (**E**) Western blot analysis of cytoplasm from RBCs. Because the G6PD gene is X linked, males were used in all experiments. G6PD_Med-_ mice are hemizygous for the knocked-in human gene; WT mice are littermate controls with the mouse WT G6PD. *n* = 3 for each group.

A targeting construct was generated to insert the human class II Mediterranean, hMed(-), variant directly into the murine G6PD locus (Figure 1A). To maintain genetic structure, the entire genomic sequence of human hMed(-) was utilized from exons 3–12. Out of concern for disrupting genomic regulatory elements in the proximal murine sequence, exons 1 and 2 and introns 1 and 2 of the murine sequence were left unaltered. As such, the final G6PD gene product is the hMed(-) form (Ser188Phe) that also has 2 amino acids in the N-terminus from the murine sequence (Figure 1B).

Out of concern that the modification may be embryonic lethal, and to allow additional experimental flexibility, the genetic construct was designed for conditional expression of the hMed(-) gene. WT mouse G6PD cDNA (exons 3–13) were inserted upstream of the human gene sequence and were flanked with *LoxP* sites. This construct is designed to express WT murine G6PD until it is exposed to CRE recombinase, at which time the mouse cDNA is excised and the human genomic sequence for the hMed(-) G6PD is expressed (Figure 1A). This mouse is called “Con-hMed(-)” and was generated using embryonic stem cells (ES) from a C57BL/6 background. Southern blot analysis was carried out both on modified ES and on founder mice to confirm the genetic alteration was made (Supplemental Figure 1; supplemental material available online with this article; https://doi.org/10.1172/jci.insight.147056DS1).

The Con-hMed(-) mouse was bred with mice that expressed germline CRE and then were crossed with WT B6 to remove the CRE gene (Figure 1A), resulting in a germline hMed(-) mouse (called G6PD_Med-_). Correct excision of the floxed region was confirmed by PCR (data not shown). Male and female G6PD_Med-_ mice were viable and fertile, and females had normal fecundity. As in humans, the murine G6PD locus is on the X chromosome; thus, heterozygous female G6PD_Med-_ mice were bred with WT males, such that 50% of the male offspring were hemizygous for hMed(-) and the other 50% were WT. In this way, G6PD_Med-_ mice were compared with littermate control WT mice.

RBCs from G6PD_Med-_ mice had only 5% of the G6PD activity compared with WT controls (Figure 1C). The decreased G6PD activity was not due to decreased gene expression because there was no difference in the amount of hMed(-) G6PD mRNA in G6PD_Med-_ compared with WT G6PD mRNA in B6 mice (Figure 1D). However, Western blot analysis of the cytoplasm from RBCs using an antibody that reacts with both human and murine G6PD demonstrated only trace G6PD in RBCs from G6PD_Med-_ mice (Figure 1E, top image), even with intentional overexpression (Figure 1E, middle image). This was not due to differences in loading the gel, as equivalent levels of β-actin were detected in all samples.

Peripheral blood was analyzed for standard metrics of RBC biology. There were no statistical differences in the values of RBC number, hemoglobin concentration, hematocrit, mean corpuscular volume, red cell distribution width, or reticulocyte counts between G6PD_Med-_ and WT mice (Supplemental Figure 2B).

### G6PD_Med-_ RBCs have decreased PPP activity ex vivo.

**Figure 2:**
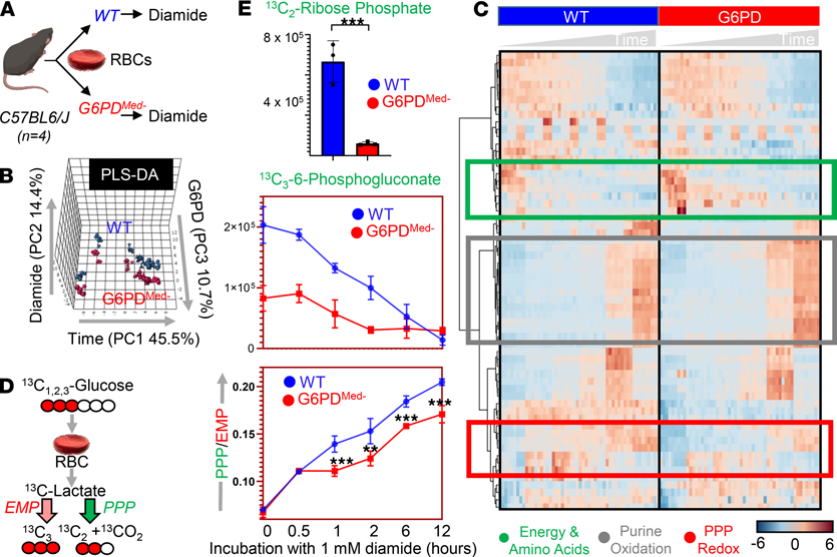
Metabolic effect of diamide challenge in RBCs from WT and G6PD_Med-_ mice. RBCs from WT and G6PD_Med-_ mice were incubated with 1 mM diamide (**A**). RBCs were tested at 0 minutes (no diamide), 30 minutes, 1 hour, 2 hours, 6 hours, and 12 hours from incubation with diamide prior to metabolomics analysis. Multivariate analyses including PLS-DA (**B**) and hierarchical clustering analysis (**C**) clearly indicate a time-dependent effect of the treatment on RBCs (PC1 explaining 45.5% of the total variance) and highlight the impact of G6PD activity (PC3 explaining 10.7% of the total variance). Significant metabolites by repeated measures 1-way ANOVA are shown in the heatmap in **C**. (**D**) The experiment was repeated by incubating RBCs in the presence of [1,2,3-^13^C_3_]-glucose. By quantifying isotopologs M+2 and M+3 of lactate (and the relative ratio), fluxes through PPP versus glycolysis can be determined (**E** — median ± range) and confirm a significantly lower activation of this pathway in RBCs from G6PD_Med-_ mice upon diamide challenge. *n* = 4 for both groups. **P < 0.01, ***P < 0.001. EMP, Embden-Meyerhof-Parnas.

**Figure 3:**
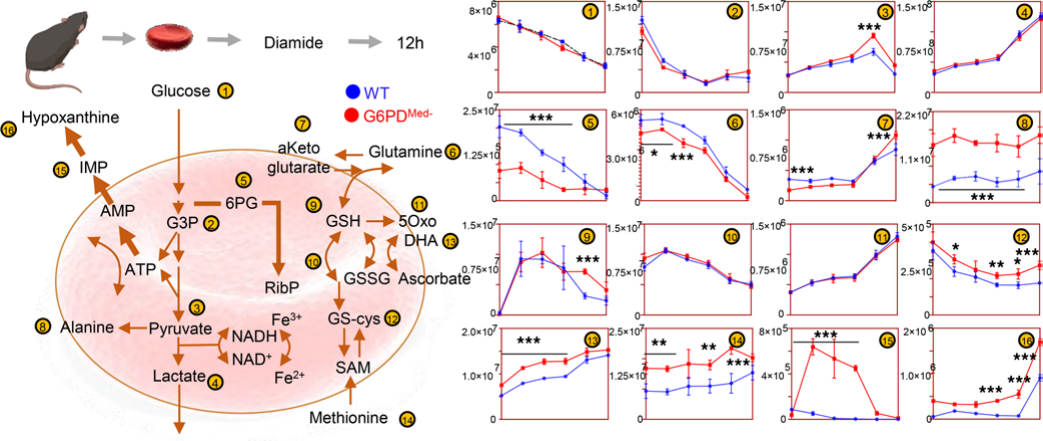
Overview of glycolysis, PPP, glutathione metabolism and recycling, and purine oxidation in RBCs from WT (shown in blue) and G6PD_Med-_ (shown in red) mouse RBCs (*n* = 3) treated with 1 mM diamide for up to 12 hours. Time points tested were 0 minutes (no diamide), 30 minutes, 1 hour, 2 hours, 6 hours, and 12 hours from incubation with diamide. Line plots indicate median ± range per each time point. *Y* axes indicate metabolite abundance in arbitrary units. *P < 0.05, **P < 0.01, ***P < 0.001.

To test how decreased G6PD activity in G6PD_Med-_ RBCs affected the PPP, RBCs from WT and G6PD_Med-_ RBCs were exposed to an oxidant challenge (1 mM diamide) for 12 hours in the presence of [1,2,3-^13^C_3_]-glucose (Figure 2A). Multivariate analyses, including partial least square-discriminant analysis (PLS-DA — Figure 2B) and hierarchical clustering analysis (Figure 2C), showed significantly distinct metabolic phenotypes between G6PD_Med-_ RBCs and WT RBCs. A version of the heatmap in Figure 2C, which also includes the top 50 significant metabolites (and isotopologs) that were significant by repeated measure ANOVA, is provided as Supplemental Figure 3. G6PD_Med-_ mice had a substantially decreased ratio of ^13^C_2_/^13^C_3_ lactate isotopologs, indicating decreased flux through the PPP (Figure 2D). Consistent with this interpretation of the decreased ratio of ^13^C_2_/^13^C_3_ lactate isotopologs, RBCs from G6PD_Med-_ mice also had significantly lower ^13^C_2_-ribose phosphate (Figure 2E), which is an end product of the oxidative phase of the PPP, as well as ^13^C_3_-phosphogluconate, which is an intermediate of the oxidative phase of the PPP. The untargeted metabolomics analyses also demonstrate that, in addition to alterations in glycolysis and PPP, G6PD_Med-_ RBCs had significantly altered redox-regulated pathways, including methionine metabolism and purine oxidation (Figure 3). As these are steady-state levels, one cannot infer changes in synthesis or consumption, only that levels are different.

### RBCs from G6PD_Med-_ mice have increased hemolysis in response in vivo to pharmacological oxidant stress.

**Figure 4:**
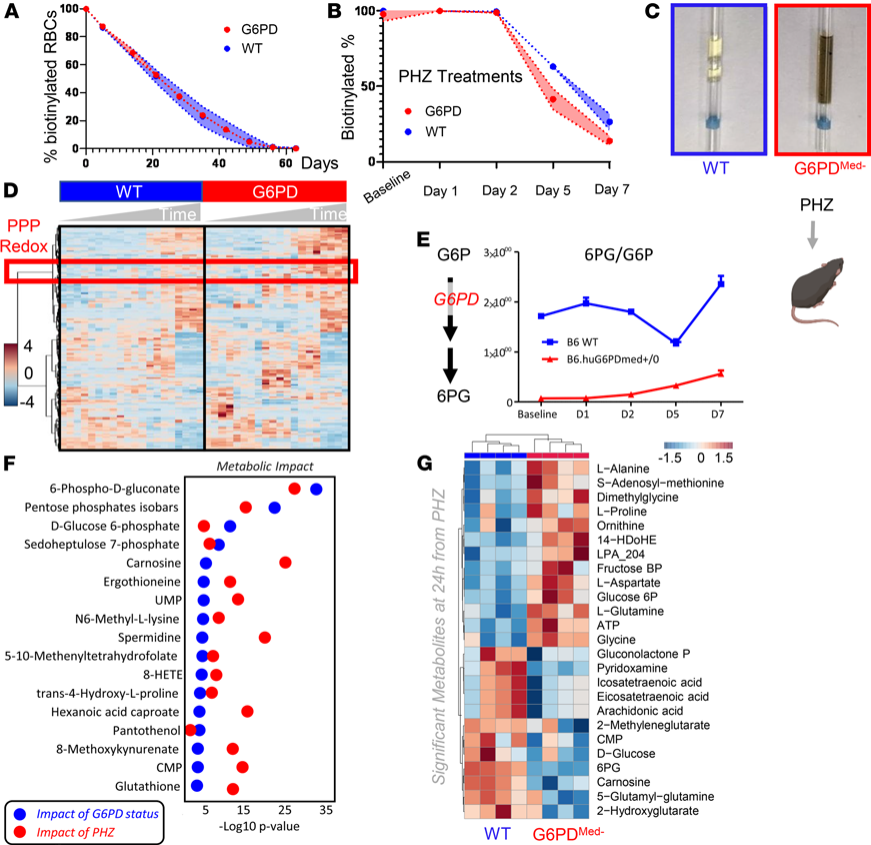
Phenylhydrazine induces brisk hemoglobinuria in G6PD-deficient mouse RBCs but not in WT. (**A**) RBC life span in mice at baseline (without any oxidative stress). (**B**) Biotinylation experiments showed significant decreases in circulating RBC survival after the second PHZ treatment in G6PD_Med-_ mice. (**C**) Hemoglobinuria was observed in G6PD_Med-._ but not WT mice after PHZ treatment. (**D**) G6PD_Med-_ RBCs at baseline and upon treatment with PHZ for up to 7 days are incapable of activating the PPP, as determined by the ratios of G6PD product/substrate, a proxy for the determination of the enzyme activity by law of mass action, as described (16) (**E** — median ± range). *Y* axis indicates glucose-6-phosphate (G6P) to 6-phospho-gluconate (6PG) ratios. *n* = 4 per group. (**F**) Pathway analysis highlighted 4 PPP-related metabolites on the top 5 significant metabolic changes by 2-way ANOVA, as a function of G6PD status (blue dots) or PHZ treatment (red dots). (**G**) Significant metabolic differences 24 hours after PHZ treatment.

To test the hypothesis that G6PD_Med-_ mice were more sensitive to oxidant stress in vivo, a method was used by which the complete blood compartment of G6PD_Med-_ mice and WT mice was each biotinylated (14). This is the equivalent of an in vivo pulse-chase experiment, allowing the determination of RBC life span without interference of erythropoiesis, because newly generated RBCs are not biotinylated. Baseline studies determined that the normal circulatory life span of G6PD_Med-_ RBCs was indistinguishable from that of WT controls (Figure 4A).

Phenylhydrazine (PHZ) is a classic chemical to induce oxidant stress in vivo and is known to cause intravascular hemolysis in human G6PD-deficient subjects (15). PHZ induced a faster rate of RBC clearance from circulation of G6PD_Med-_ mice compared with WT controls (Figure 4B). In addition, G6PD_Med-_ but not WT controls displayed hemoglobinuria (Figure 4C). Metabolomics analyses were performed in blood from the mice at baseline (prior to biotinylation), at day 1 (after biotinylation), at day 2 (middle of PHZ challenge), and at days 5 and 7 (after PHZ challenge). Results are summarized in the heatmap in Figure 4D, with more extensive data provided in tabulated and vectorial forms (including metabolite names) in Supplemental Figure 4.

**Figure 5:**
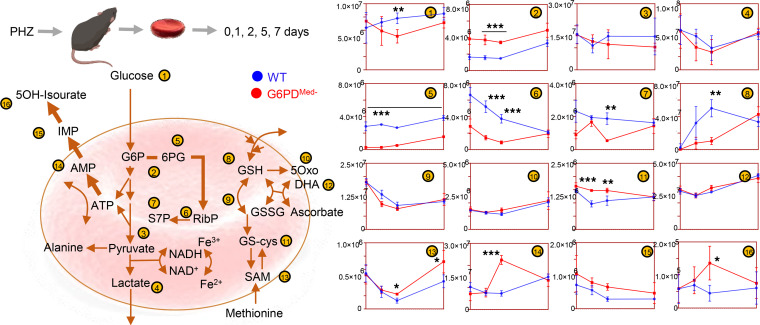
Metabolic impact of PHZ treatment in vivo on WT and G6PD-deficient mouse RBCs. Overview of glycolysis, PPP, glutathione metabolism and recycling, and purine oxidation in RBCs from WT (blue) and G6PD_Med-_ (red) mice (*n* = 3) treated with 1 mM PHZ for up to 7 days. Time points tested were 0 (no PHZ), 1, 2, 5, and 7 days from treatment with PHZ. Line plots indicate median ± range per each time point. *Y* axes indicate metabolite abundance in arbitrary units. *P < 0.05, **P < 0.01, ***P < 0.001.

The ratio of steady-state glucose-6-phosphate (G6P) to 6-phospho-gluconate (6PG) (i.e., the substrate and downstream metabolite of G6PD, respectively) is a proxy for G6PD activity (16) (Figure 4E). Consistent with the ex vivo measurements described above, in vivo measurements of 6PG/G6P ratios were significantly lower at baseline and following PHZ exposure in G6PD_Med-_ mice, compared with WT animals (Figure 4E). Repeated measure ANOVA of time course data further highlighted that the top 4 metabolites that significantly differed between the 2 groups as a function of PHZ stimulation were PPP metabolites (i.e., G6P, 6PG, ribose phosphate and isobaric isomers, sedoheptulose phosphate; Figure 4F). Other metabolites related to redox homeostasis were significantly affected by PHZ stimulation, as a function of G6PD status, including glutathione, oxylipins (hydroxyeicosatetraenoic acids), proteolysis markers (e.g., hydroxyproline, methyl-lysine), and other antioxidant metabolites (e.g., carnosine, ergothioneine; Figure 4E, Supplemental Figure 4). These changes between G6PD_Med-_ and WT mice were most evident after PHZ injection (a heatmap of the top 25 significant metabolites by unpaired *t* test for this time point is provided in Figure 4G). A detailed representation of these metabolites and related pathways as a function of the entire time course is provided in the form of line plots in Figure 5.

### RBCs from G6PD_Med-_ mice have normal posttransfusion recovery after storage.

**Figure 6:**
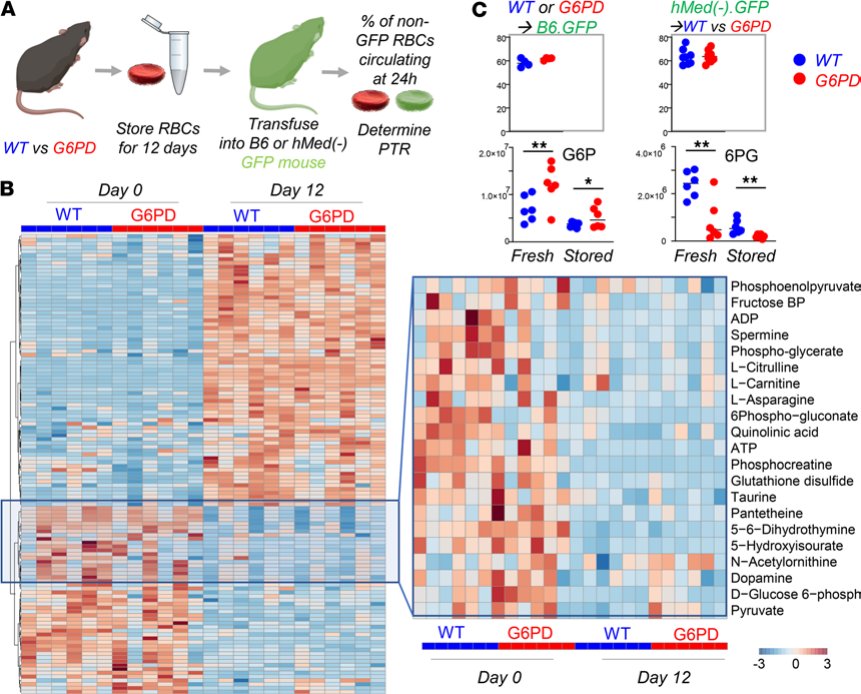
G6PD-deficient mouse RBCs have comparable metabolism and posttransfusion recovery to WT RBCs at the end of storage. RBCs from WT and G6PD_Med-_ mice (*n* = 6) were stored under conditions mimicking storage in the blood bank for 12 days (**A**). At the end of storage, RBCs were transfused into UbiC-GFP C57BL/6-Tg(UBC-GFP)30Scha/J (Ubi-GFP) mice, and flow cytometry studies were performed to determine the percentage of transfused RBCs circulating at 24 hours from transfusion, which was determined to be comparable between the 2 groups. Transfusing G6PD_Med-_ GFP into WT or G6PD_Med-_ non-GFP recipients did not impact the posttransfusion recovery (PTR) percentage measurement. (**B**) Metabolic phenotypes of G6PD_Med-_ RBCs showed some significant differences at baseline (especially with respect to glycolysis, the PPP, and glutathione homeostasis). However, these changes were not appreciable by the end of storage. (**C**) Individual data points for G6P and 6PG are shown (significance calculated by Mann-Whitney unpaired *t* test). *P < 0.05, **P < 0.01.

Refrigerated storage of human RBCs is an iatrogenic source of oxidant stress that is a necessary logistical component of blood banking and clinical transfusion practice. It was recently reported that when RBCs are stored from humans with G6PD deficiency and are then transfused, they have a modest (i.e., ~6%), but statistically significant, decrease in 24-hour posttransfusion recovery. To test how the hMed(-) mutation affects storage of murine RBCs, donor mice were exsanguinated, and RBCs were stored using an established methodology that models the human setting (17). In this case, the 24-hour posttransfusion recovery was the same for G6PD_Med-_ and WT mice (Figure 6A).

The human studies that demonstrated decreased 24-hour recoveries of transfused RBCs from G6PD-deficient donors used autologous transfusions of radiolabeled RBCs; as such, the G6PD-deficient RBCs were not only exposed to the oxidative stress of storage but also introduced into a recipient with potentially altered redox biology due to G6PD deficiency. To allow for autologous transfusion studies in mice, G6PD_Med-_ mice were crossed with B6.GFP mice. RBCs from G6PD_Med-_ mice were then collected, stored, and transfused into either B6.GFP mice with normal G6PD or into G6PD_Med-_ GFP recipients; in this approach, transfused RBCs are enumerated as the GFP-negative population. No difference in 24-hour posttransfusion recovery was observed in G6PD_Med-_ GFP, as compared with B6.GFP recipients, ruling out that differences in storage biology would be present if both donor and recipient were G6PD_Med-_ (Figure 6A).

Stored RBCs were also analyzed by metabolomics. At baseline (i.e., before storage), differences were observed in glycolysis and the PPP, glutathione homeostasis, and purine and amino acid metabolism (Figure 6B); specifically, genotype-dependent alterations were detected with regard to arginine, tryptophan, and tyrosine metabolism, as gleaned by differential levels of citrulline, indoles, and dopamine, respectively (Figure 6B — right panel). Although increases in G6P and decreases in 6PG were significant in G6PD_Med-_ mice both at baseline and at the end of storage (12 days), at day 12 the fold changes for both metabolites between the 2 genotypes were negligible (Figure 6C).

### Effects of hMed(-) on nonerythroid tissues.

**Figure 7:**
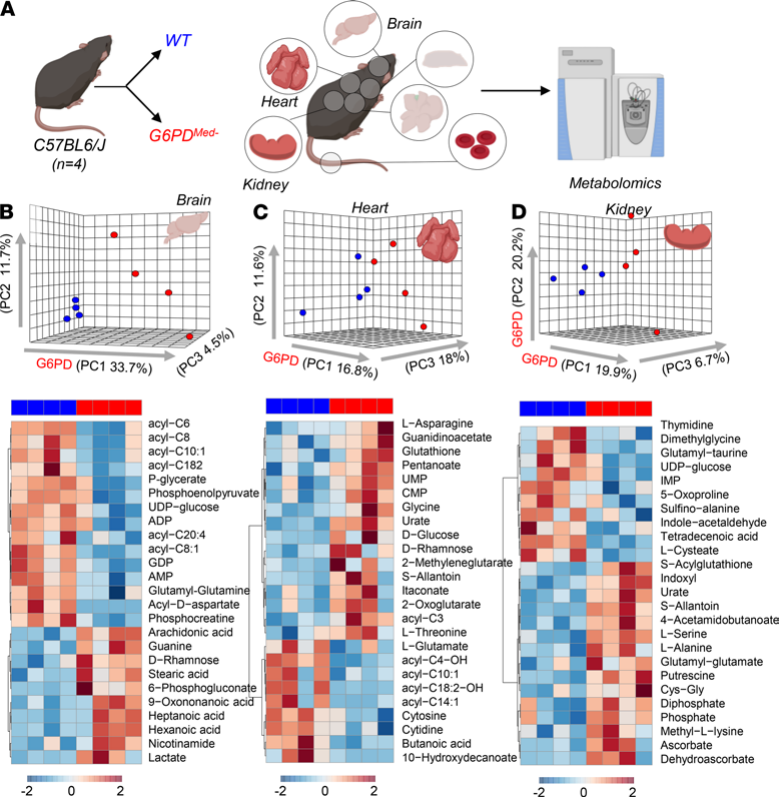
Metabolic analyses of brain, heart, and kidney from WT and G6PD_Med-_ mice. (**A**) Significant metabolic changes were observed in all the organs tested from transgenic mice, compared with WT controls, *n* = 4 for both groups. (**B**–**D**) as highlighted by multivariate principal component and hierarchical clustering analyses (only the top 25 significant metabolites by *t* test are shown).

**Figure 8:**
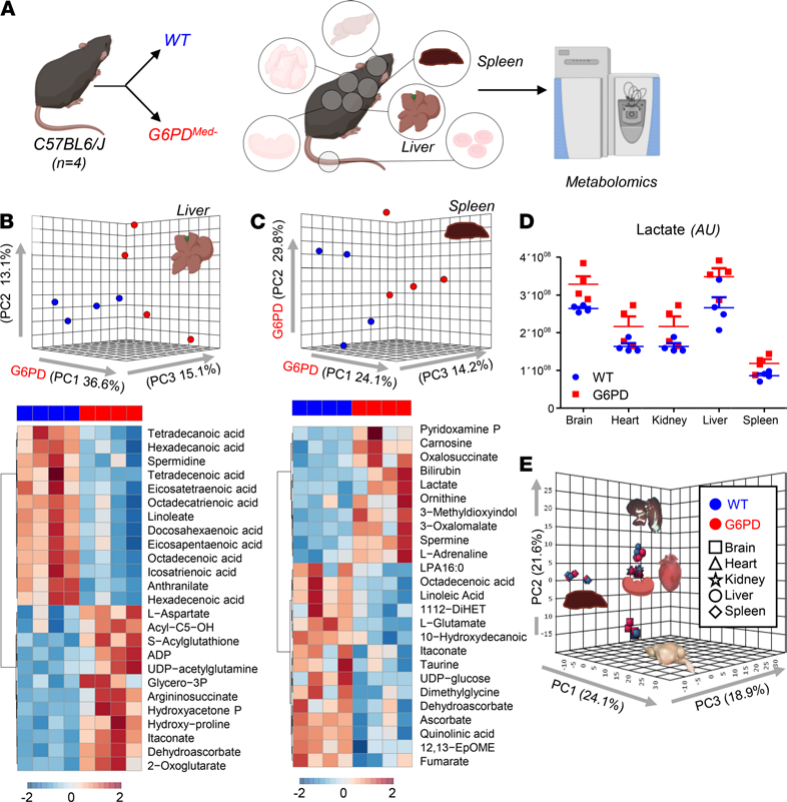
Metabolic analyses of liver and spleen from WT and G6PD_Med-_ mice. (**A**) Significant metabolic changes were observed in all the organs tested from G6PD_Med-_ mice, compared with WT controls, (**B** and **C**) as highlighted by multivariate principal component and hierarchical clustering analyses (only the top 25 significant metabolites by *t* test are shown). The majority of metabolic changes in organs of G6PD_Med-_ mice are related to fatty acid metabolism and acyl-carnitines, followed by amino acid metabolism and glycolysis. Lactate levels were significantly lower in all organs of G6PD_Med-_ mice. *n* = 4 for both groups. (**D**) Individual data points are shown for lactate in each organ in WT (blue bars) and G6PD_Med-_ (red bars). (**E**) Organ-to-organ metabolic differences overweighed differences between WT and G6PD_Med-_ mice.

G6PD deficiency has often been considered RBC specific, because, unlike RBCs that cannot synthesize protein, nucleated cells can compensate for decreased G6PD enzyme activity by increasing synthesis. However, in humans with G6PD deficiency, other tissues (e.g., muscle and endothelial cells in the pulmonary artery) have decreased G6PD activity, albeit not as severe as seen in RBCs (18, 19). To perform a detailed analysis of multiple organs, which is not feasible in human studies, metabolomics analyses were performed on brain, heart, kidney, liver, and spleen of G6PD_Med-_ compared with WT mice (Figure 7 and Figure 8). Detailed results from hierarchical clustering analyses of all the metabolomics data are provided for each organ: Supplemental Figures 5 (liver), 6 (brain), 7 (heart), 8 (kidney), 9 (spleen), and 10 (merged organs). An overview of the multivariate analyses, including PLS-DA and heatmaps reporting the top 25 significant metabolites by unpaired *t* test, is provided for each organ in Figures 7 and 8.

Of note, significant decreases (*P* < 0.05) in carnitine-conjugated fatty acids and increases in free fatty acids were noted in all tissues of G6PD_Med-_ mice, most notably in high-oxygen-consuming organs, such as the brain (18.4%), heart (11.6%), and liver (20.4%). There was a distinct change in β-oxidation of unsaturated fatty acids, a hallmark of oxidative metabolism and an NADPH-dependent process (20), suggesting a potential mechanistic linkage between a reduced capacity to sustain NADPH generation via the PPP in G6PD_Med-_ mice and altered fatty acid metabolism. Similarly, several organs exhibited decreased levels of high-energy phosphate compounds (e.g., ADP, AMP, GDP, phosphocreatine), increased levels of amino acids (metabolized in mitochondria), and increased levels of carboxylic acids (e.g., 2-oxoglutarate and itaconate), further suggesting depressed mitochondrial metabolism. Consistent with an apparent depression of oxidative metabolism, all organs also exhibited increased levels of lactate. However, multivariate analyses indicated that — in the absence of oxidant stress — the metabolic differences among organs were more pronounced than the metabolic differences observed within the same organ in G6PD_Med-_ and WT mice.

## Discussion

Herein we describe a murine model of G6PD deficiency that overcomes the limitations of existing models and has allowed an exploration into metabolomics of both RBCs and solid organs. These data provide potentially novel insight into the effects of an enzymopathy that affects approximately 5% of all humans. The extent of the decreased activity in G6PD_Med-_ mice, as compared with WT, approximated what is seen in humans (i.e., 5%–10% of normal activity), and, as is the classic finding in humans, pharmacological oxidative stress induced brisk hemolysis in vivo with hemoglobinuria. Metabolic tracing experiments demonstrated a significant decrease in glucose flux through the PPP and increased sensitivity to pharmacological oxidative stress. Although detailed metabolomics of sold organs in humans with G6PD deficiency has not been reported to our knowledge, the decreased PPP in organs from the G6PD_Med-_ mouse is consistent with known mild decreases in G6PD-deficient organs in humans (21–25).

Human G6PD-Med(-) subjects have a mildly shortened RBC circulatory life span, i.e., 120 days in G6PD-normal controls, as compared with 100 days in G6PD-Med(-) subjects (26). In contrast, RBCs from the hMed(-) mice had a normal RBC circulatory life span. It can be speculated that the murine RBC life span (55 days) may not be long enough for the defects to accumulate, as they do in humans over 120 days. It is also important to note that, in their normal state, mice have higher glutathione (GSH) levels than do humans, which may also mitigate the effects of G6PD deficiency in the absence of external oxidative challenge. Alternatively, other pathways involved in GSH synthesis (e.g., ascorbate synthesis) exist in mice but not in humans (27).

To our knowledge, this report contains the first metabolomic analysis of RBC changes after exposure to PHZ (in either WT or G6PD-deficient mice); however, one caveat with this is that the changes could reflect metabolites in newly formed reticulocytes versus alterations in RBCs at the time of treatment. In vitro analysis of diamide-treated RBCs does not suffer this issue as new RBC generation is absent. Moreover, detailed metabolomics have been reported following in vitro exposure of human G6PD-deficient (G6PD-def) RBCs to diamide (28). In particular, it was reported that G6PD-def RBCs (but not normal RBCs) responded to diamide though depletion of GSH through oxidation (e.g., decreased GSH with increasing glutathione disulfide). In response, GSH synthesis was increased as evidenced by decreases in GSH precursors (e.g., γ-glutamylcysteine) and generation of byproducts of GSH synthesis (e.g., ophthalmate). ATP was also decreased in G6PD-def RBCs, which was interpreted as reflecting consumption of ATP by GSH synthesis. This resulted in a substantial increase in glycolytic metabolism in G6PD-def RBCs, presumably to compensate for the energy depletion of ATP consumption; however, the glycolysis was abnormal in that pyruvate levels did not increase, suggestive of oxidative deactivation of pyruvate kinase. As expected, downstream metabolites of the PPP were decreased in G6PD-def RBCs. We also showed that the increase in glycolysis of G6PD-def RBCs in response to diamide correlated with increased AMPK activity that was associated with increased AMP levels due to ATP consumption.

Consistent with the above-cited studies, when we exposed G6PD_Med-_ mouse RBCs to diamide, we observed significant alterations (*P* < 0.05) of GSH metabolism, accompanied by the inability to preserve nondeaminated high-energy purine pools in the face of decreased PPP activity. This is relevant in that RBC purine deamination by oxidant stress–activated AMP deaminase 3 is a hallmark of impaired energy metabolism, ultimately associated with a decreased ability of the RBC to circulate (29) following oxidant insult and an overall shorter RBC life span in mice that are unable to replenish the ATP pool (e.g., mice with altered AMPK activity) (30). Of note, increased purine deamination by AMP deaminase 3 activation phenocopies the protective effect of G6PD deficiency on malaria infection (31). However, a number of specific metabolic differences were also observed between diamide responses of G6PD_Med-_ mouse RBCs and that reported with human RBCs. Surprisingly, GSH levels did not decrease in mouse G6PD_Med-_ RBCs in response to diamide treatment; however, GSH levels did decrease in response to other oxidative stress (e.g., PHZ). It is worth noting that in the human studies, only a small number of humans were studied, they had a different G6PD mutation (Canton and not Mediterranean), and there may also be methodological differences (28). Future study will be required to assess the meaning of these respective biologies.

It was recently reported that when stored under blood bank conditions, RBCs from G6PD-def humans have decreased posttransfusion recovery (32). However, the decrease in posttransfusion recovery was modest (~6%). Importantly, most subjects in this study had the milder A- form of G6PD deficiency. Only a single subject was of the G6PD-Med(-) type, and unexpectedly, that subject had a posttransfusion recovery overlapping with the median measurement from the control group. The basis for this is unclear, but it indicates that the current findings with the G6PD_Med-_ mouse are not in conflict with the one human G6PD-Med(-) subject who has been studied. There are conflicting reports regarding the extent to which G6PD activity decreases in RBC storage; it is unclear if these differences were due to differing populations or methodologies (e.g., differences in storage additives such as SAGM vs. AS-3) (33, 34). However, changes in metabolism from the stored RBCs from the G6PD_Med-_ mice are largely consistent with larger metabolic studies on RBCs from human donors with the G6PD-Med(-) variant (e.g., dopamine and pyruvate/lactate ratio); although posttransfusion recoveries were not reported for this group (35). Finally, recent findings from the Recipient Epidemiology and Donor Evaluation Study-III showed similar findings during storage of units from G6PD-def donors, although these were largely of the A- variant (36).

Although studies outside the RBC compartment in G6PD-def humans are limited, decreased G6PD activity in nonerythroid compartments has been reported, including in leukocytes (24), platelets (25), liver (23), and muscle (21, 22). Similar to what has been reported in humans, we show modest decreases in PPP activity in multiple organs, consistent with a mild decrease in G6PD activity. The less severe deficiency in nonerythroid organs is presumably because, unlike RBCs, they have ongoing synthesis of the destabilized enzyme. More importantly, we provide a comprehensive metabolic description of multiple organs from G6PD-def mice, showing a role for altered PPP activation in the cross-regulation of several NADPH-dependent pathways in nonerythroid tissues. Most importantly, the current data highlight a G6PD deficiency–dependent alteration in fatty acid amounts, unsaturation, and metabolism (as gleaned by the levels of free and carnitine-conjugated fatty acids). Notably, fatty acid synthesis (a key anabolic requirement for rapidly proliferating cells), desaturation, and catabolism are NADPH-dependent processes.

The role of NADPH-dependent lipid metabolism is being increasingly appreciated in tumor biology (37). Thus, our observations, combined with the appreciation of the high penetration of G6PD deficiency in human populations, raise the possibility of a previously unappreciated role for G6PD status and biology in solid tumors, analogous to what has been proposed for hematological malignancies (38). In addition, our observations suggest an indirect role for G6PD status on the capacity to metabolize fatty acids in high-oxygen-demand organs (e.g., brain, heart, liver), making this model potentially useful for the study of how G6PD deficiency may affect other disorders that have been associated with G6PD deficiency in humans, including kidney disease and diabetes (7), pulmonary arterial hypertension (9), amyotrophic lateral sclerosis, Huntington disease, Parkinson disease, Alzheimer disease (8), and multiple sclerosis (39).

In summary, we report a model of G6PD deficiency in mice using a humanized enzyme of the Med(-) variant and describe metabolic findings with regard to normal RBC biology, oxidant stress responses, and systemic alterations in peripheral organs. This model promises to be of high utility in ongoing studies in a wide variety of biologies and pathologies in which G6PD deficiency has been implicated, e.g., aging (40), pulmonary hypertension, neurological disorders (8), exercise physiology, antimalarial toxicology (41), and mechanisms of genetic resistance to malarial infection.

## Methods

### Mice.

The Con-hMed(-) were generated, as described in detail in the Results section, using Bruce4 ES. Con-hMed(-) mice were bred to mice expressing CRE under a CMV promoter [The Jackson Laboratory mouse B6.C-Tg(CMV-cre)1Cgn/J stock 006054], resulting in G6PD_Med-_ mice, which were maintained through backcrossing to C57BL/6J mice. Female heterozygous G6PD_Med-_ mice were bred with WT C57BL/6J mice such that all WT and G6PD_Med-_ mice were littermate controls from the same breeding colony. Male mice were used for all experiments unless otherwise noted. Ubi-GFP [UbiC-GFP C57BL/6-Tg(UBC-GFP)30Scha/J stock 004353] and C57BL/6J mice were purchased from The Jackson Laboratory and bred as described. All experiments were carried out under an IACUC-approved protocol at either BloodworksNW or the University of Virginia. All mice were used from 2–6 months of age — for any given experiment mouse ages were matched.

### RNA isolation/real-time PCR.

Bone marrow RNA was isolated using TRIzol (Thermo Fisher Scientific). RNA was converted to cDNA using the iScript gDNA Clear kit (Bio-Rad). Real-time quantitative PCR was performed on a QuantStudio 6 Flex (Applied Biosystems, Thermo Fisher Scientific), using the following TaqMan primers and probes: ActB (Mm00607939_s1), Pol2Ra (Mm00839502_m1), murine G6PDx (Mm04260097_m1; spans exons 1–2), murine G6PDx (Mm00656735_g1; spans exons 12–13), human G6PD (Hs00959072_g1; spans exons 3–4), and human G6PD (Hs00959073; spans exons 4–5). Controls lacking reverse transcriptase were run for every RNA preparation to control for contaminating genomic DNA; no samples lacked reverse transcriptase amplification (data not shown). Data were analyzed with QuantStudio 6 software using relative quantitation (ΔΔCt), with ActB as the normalization factor and B6 as the control strain.

### RBC lysis and hemoglobin depletion.

RBC ghosts (membranes) were prepared by hypotonic lysis, followed by depletion of hemoglobin through 2 rounds of treatment with Hemoglobind from Biotech Support Group, followed by centrifugation. Supernatants were used for Western blotting (below).

### Western blotting.

Hemoglobin-depleted supernatants were electrophoresed on 4%–12% NuPAGE Bis-Tris gels (Thermo Fisher Scientific) under reducing conditions and transferred to PVDF membranes. As a control, recombinant human G6PD protein (Abcam, catalog NP0007) was also included. Membranes were blocked in 2.5% milk/2.5% BSA in TBS-Tween, probed with rabbit anti-human/mouse G6PD (Abcam, catalog EPR20688) or rabbit anti-actin (Cell Signaling Technology, catalog 4970), and imaged by standard chemiluminescence techniques on an ImageQuant 800 (Cytiva).

### G6PD activity assay.

For the quantitative determination of G6PD activity, the Glucose-6-Phosphate Dehydrogenase Reagent Set was used (Pointe Scientific, catalog G7583180). Following the manufacturer’s protocol, 10 μL of whole unwashed RBCs were assayed using a heated cuvette spectrophotometer (Nanophotometer C40, Implen); hemoglobin (g/dL) was measured using approximately 70 μL whole, unwashed RBCs on an ABLX90 (Radiometer). Results are presented as G6PD activity (U/g hemoglobin).

### Blood storage and transfusion studies.

Blood storage and transfusion and determination of posttransfusion recovery were carried out as described in detail in previous studies (17, 42).

### RBC life span determination and oxidant stress challenge.

RBC life span determination was carried out by the biotinylation method (14) and as described in detail in previous studies (43). PHZ was given to mice through 3 intraperitoneal injections of 0.01 mg/g administered 12 hours apart.

### Sample processing and metabolite extraction for ultra-high-pressure liquid chromatography–mass spectrometry.

A total of 50 μL of frozen RBC aliquots or 10 mg of snap-frozen organ tissues were extracted in 450 μL or 1 mL, respectively, of ice-cold methanol/acetonitrile/water (5:3:2 *v/v*). Samples were agitated at 4°C for 30 minutes followed by centrifugation at 10,000*g* for 10 minutes at 4°C, as described (44). Protein pellets were discarded, and supernatants were stored at –80°C prior to metabolomic analysis.

### Ultra-high-pressure liquid chromatography–mass spectrometry metabolomics.

Samples were randomized and 10 μL aliquots of extracts were injected using an ultra-high-pressure liquid chromatography (UHPLC) system (Vanquish, Thermo Fisher Scientific) and run on a Kinetex C18 column (150 *×* 2.1 mm, 1.7 μm — Phenomenex) at 250 μL/min (isocratic: 5% Optima acetonitrile, 95% Optima H_2_O, 0.1% formic acid) (45) and 400 μL/min (5 or 17 minutes gradient 5%–95% B; A: water + 0.1% formic acid, B: acetonitrile + 0.1% formic acid) (46, 47). Formic acid in mobile phases was replaced by 1 mM ammonium acetate for negative-mode runs. The UHPLC system was coupled online with a Q Exactive mass spectrometer (Thermo Fisher Scientific), scanning in Full MS mode (2 μscans) at 70,000 resolution in the 60–900 *m/z* range operated in either polarity mode. Eluates were subjected to electrospray ionization in positive and negative ion modes (separate runs) with 4 kV spray voltage, 15 psi sheath gas, and 5 psi auxiliary gas. Separate Top15 ddMS2 runs were performed on technical mixes composed of 10 μL of each extract from each one of the samples in the batch. Chromatographic and mass spectrometry technical stability were assessed by determining coefficients of variation less than 10% for metabolites in mixed controls run every 5 injections in the queue. MS1 and data-dependent MS2 acquisition (48), data analysis, and elaboration were performed, as described (46, 47).

### Statistics.

Graphs and statistical analyses (either unpaired, 2-tailed *t* test or repeated measure or 2-way ANOVA) were generated with GraphPad Prism 8.1.2 (GraphPad Software, Inc), GENE E (Broad Institute), and MetaboAnalyst 4.0 (49). Real-time PCR was analyzed using 2-way ANOVA with Sidak’s post hoc testing, which revealed no significant differences in mRNA levels between WT and G6PD_Med-_ mice for either Pol2RA or Mm04260097_m1.

### Study approval.

All experiments were carried out under IACUC-approved protocols at BloodworksNW or University of Virginia.

## Author contributions

ADA, SLS, EAH, ROF, EFS, MK, TT, and JCZ conceived of the studies, designed experiments, and interpreted data. HLH, AMH, EFS, BCB, MJW, and XF carried out experiments, generated data, and interpreted data. ADA, HLH, AMH, KHD, BCB, MJW, MK, EFS, SLS, EAH, ROF, XF, TT, and JCZ were actively involved in the writing of the manuscript and the interpretation of data.

## Supplementary Material

Supplemental data

## Version 1 06/17/2021

In-Press Preview

## Version 2 07/22/2021

Electronic publication
