# Supplementary material for: Hematologic and systemic metabolic alterations due to Mediterranean class II G6PD deficiency in mice
Source: JCI Insight. 2021 Jul 22;6(14):e147056. doi: 10.1172/jci.insight.147056 (PMC8410095; doi:10.1172/jci.insight.147056)
Supplement: Supplemental data [file jciinsight-6-147056-s211.pdf]

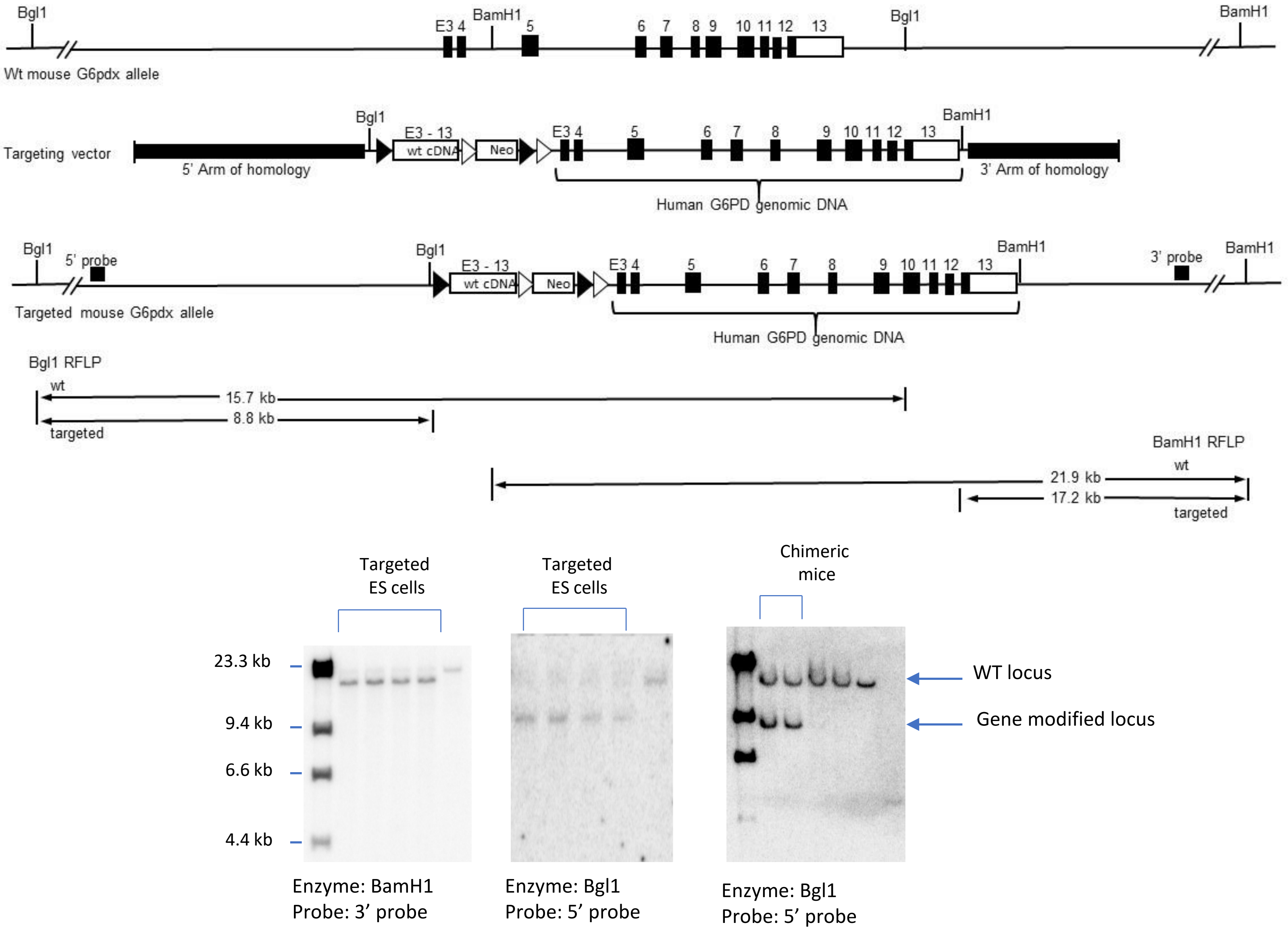

Supplemental Figure 1. The schematic shows restriction sites in wild-type (Wt) and gene modified loci. Southern blot analysis of ES cells and the final mice demonstrate homologous recombination and the absence of any random integration.

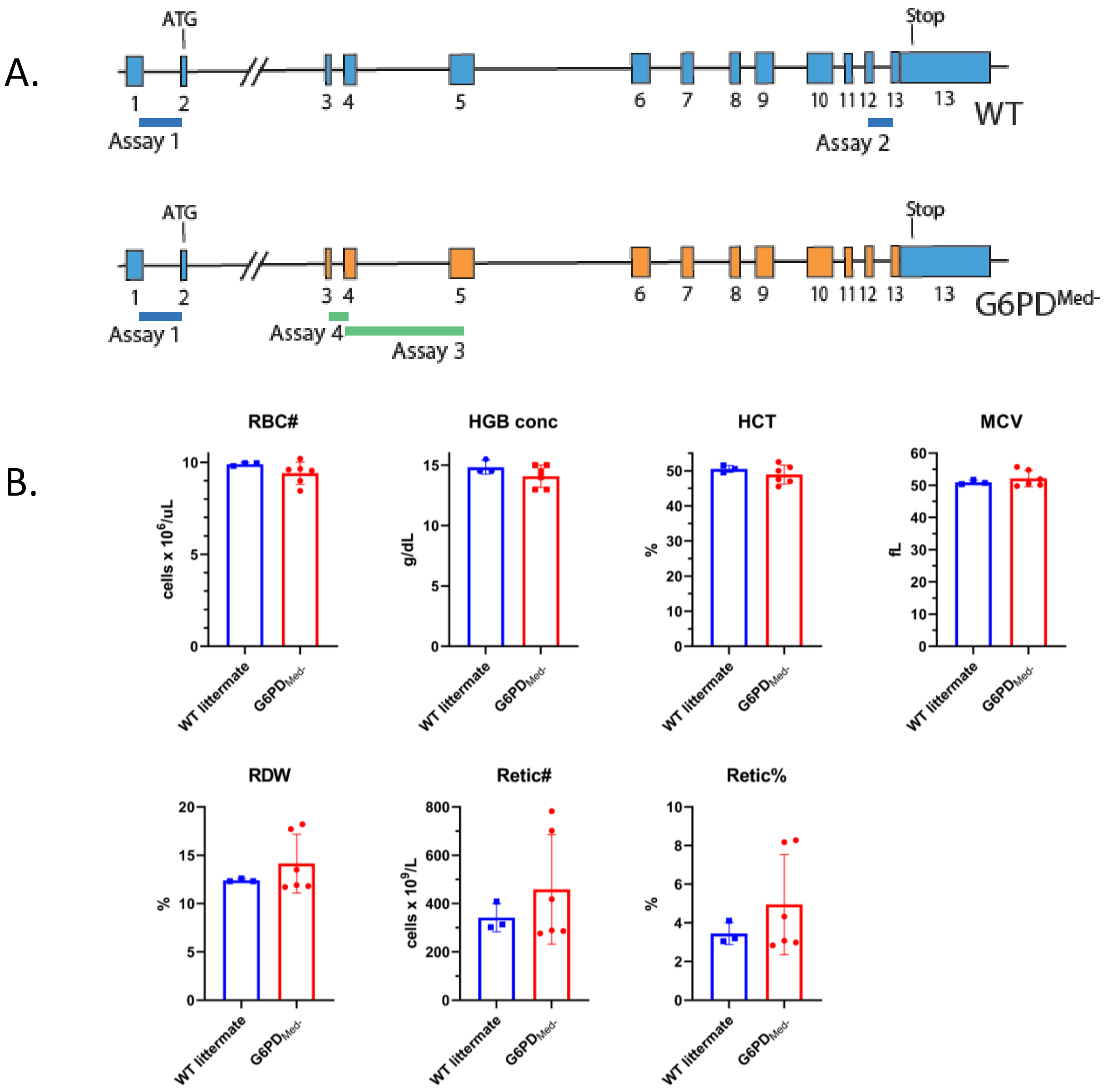

Supplemental Figure 2. (A) Schematic of wild-type (WT) and $G6PD_{Med-}$ Genes indicating the location of allele specific probes. **(B)** Values of indicated blood parameters. All bar graphs represent means with SD, no significant differences were observed by Welch's t-test (assumption of Gaussian distribution and unequal SDs)

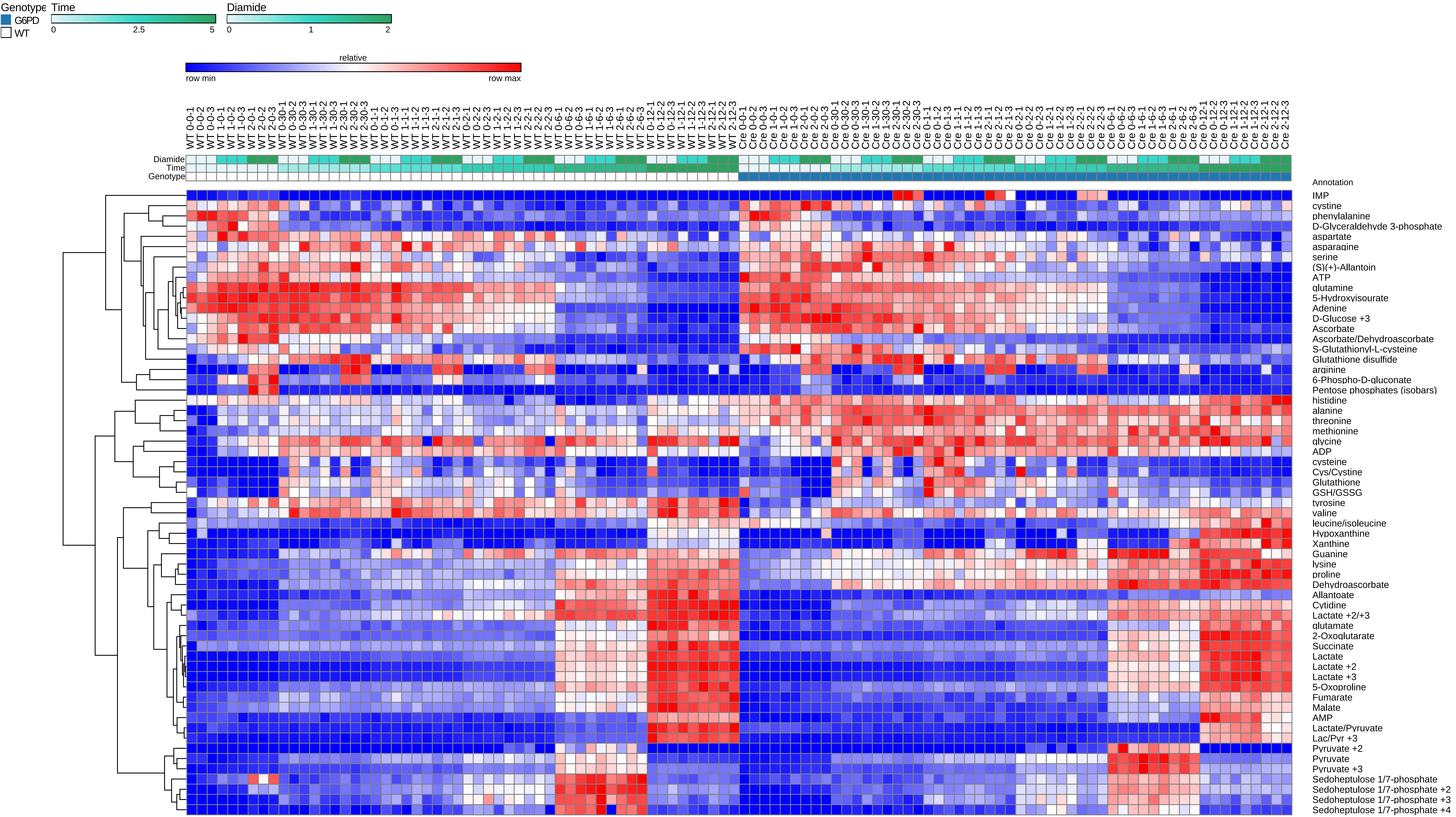

Genotype
G6PD
WT
Time
0
2.5
5
Diamide
0
1
2
relative
row min
row max
WT 0-0-1
WT 0-0-2
WT 0-0-3
WT 1-0-1
WT 1-0-2
WT 1-0-3
WT 2-0-1
WT 2-0-2
WT 2-0-3
WT 0-30-1
WT 0-30-2
WT 0-30-3
WT 1-30-1
WT 1-30-2
WT 1-30-3
WT 2-30-1
WT 2-30-2
WT 2-30-3
WT 0-1-1
WT 0-1-2
WT 0-1-3
WT 1-1-1
WT 1-1-2
WT 1-1-3
WT 2-1-1
WT 2-1-2
WT 2-1-3
WT 0-2-1
WT 0-2-2
WT 0-2-3
WT 1-2-1
WT 1-2-2
WT 1-2-3
WT 2-2-1
WT 2-2-2
WT 2-2-3
WT 0-6-1
WT 0-6-2
WT 0-6-3
WT 1-6-1
WT 1-6-2
WT 1-6-3
WT 2-6-1
WT 2-6-2
WT 2-6-3
WT 0-12-1
WT 0-12-2
WT 0-12-3
WT 1-12-1
WT 1-12-2
WT 1-12-3
WT 2-12-1
WT 2-12-2
WT 2-12-3
Cre 0-0-1
Cre 0-0-2
Cre 0-0-3
Cre 1-0-1
Cre 1-0-2
Cre 1-0-3
Cre 2-0-1
Cre 2-0-2
Cre 2-0-3
Cre 0-30-1
Cre 0-30-2
Cre 0-30-3
Cre 1-30-1
Cre 1-30-2
Cre 1-30-3
Cre 2-30-1
Cre 2-30-2
Cre 2-30-3
Cre 0-1-1
Cre 0-1-2
Cre 0-1-3
Cre 1-1-1
Cre 1-1-2
Cre 1-1-3
Cre 2-1-1
Cre 2-1-2
Cre 2-1-3
Cre 0-2-1
Cre 0-2-2
Cre 0-2-3
Cre 1-2-1
Cre 1-2-2
Cre 1-2-3
Cre 2-2-1
Cre 2-2-2
Cre 2-2-3
Cre 0-6-1
Cre 0-6-2
Cre 0-6-3
Cre 1-6-1
Cre 1-6-2
Cre 1-6-3
Cre 2-6-1
Cre 2-6-2
Cre 2-6-3
Cre 0-12-1
Cre 0-12-2
Cre 0-12-3
Cre 1-12-1
Cre 1-12-2
Cre 1-12-3
Cre 2-12-1
Cre 2-12-2
Cre 2-12-3
Diamide
Time
Genotype
Annotation
IMP
cystine
phenylalanine
D-Glyceraldehyde 3-phosphate
aspartate
asparagine
serine
(S)(+)-Allantoin
ATP
glutamine
5-Hydroxyisourate
Adenine
D-Glucose +3
Ascorbate
Ascorbate/Dehydroascorbate
S-Glutathionyl-L-cysteine
Glutathione disulfide
arginine
6-Phospho-D-gluconate
Pentose phosphates (isobars)
histidine
alanine
threonine
methionine
glycine
ADP
cysteine
Cys/Cystine
Glutathione
GSH/GSSG
tyrosine
valine
leucine/isoleucine
Hypoxanthine
Xanthine
Guanine
lysine
proline
Dehydroascorbate
Allantoate
Cytidine
Lactate +2/+3
glutamate
2-Oxoglutarate
Succinate
Lactate
Lactate +2
Lactate +3
5-Oxoproline
Fumarate
Malate
AMP
Lactate/Pyruvate
Lac/Pyr +3
Pyruvate +2
Pyruvate
Pyruvate +3
Sedoheptulose 1/7-phosphate
Sedoheptulose 1/7-phosphate +2
Sedoheptulose 1/7-phosphate +3
Sedoheptulose 1/7-phosphate +4

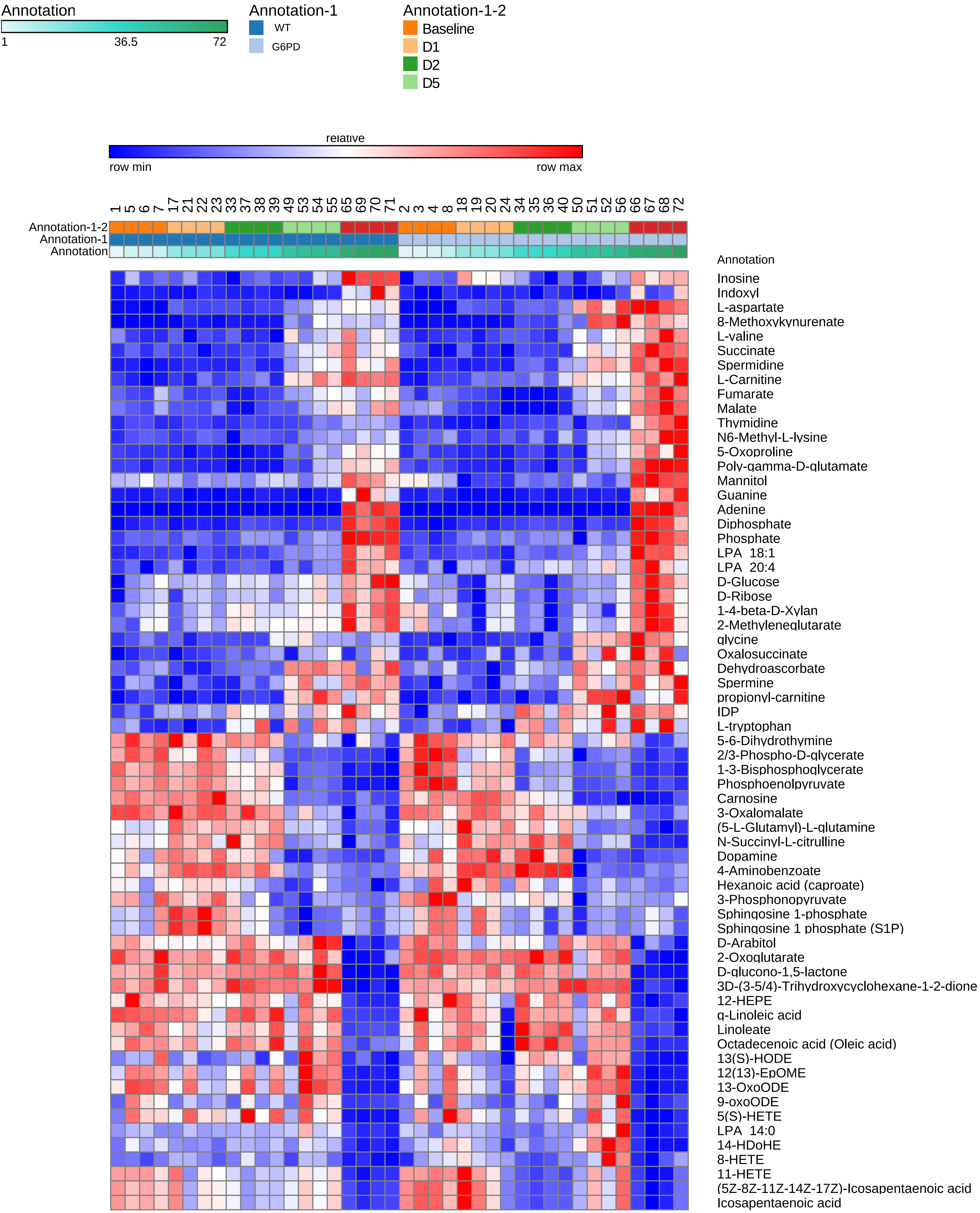

Annotation
1
36.5
72
Annotation-1
WT
G6PD
Annotation-1-2
Baseline
D1
D2
D5
relative
row min
row max
1 5 6 7 17 21 22 23 33 37 38 39 49 53 54 55 65 69 70 71 2 3 4 8 18 19 20 24 34 35 36 40 50 51 52 56 66 67 68 72
Annotation-1-2
Annotation-1
Annotation
Annotation
Inosine
Indoxyl
L-aspartate
8-Methoxykynurenate
L-valine
Succinate
Spermidine
L-Carnitine
Fumarate
Malate
Thymidine
N6-Methyl-L-lysine
5-Oxoproline
Poly-gamma-D-glutamate
Mannitol
Guanine
Adenine
Diphosphate
Phosphate
LPA 18:1
LPA 20:4
D-Glucose
D-Ribose
1-4-beta-D-Xylan
2-Methyleneglutarate
glycine
Oxalosuccinate
Dehydroascorbate
Spermine
propionyl-carnitine
IDP
L-tryptophan
5-6-Dihydrothymine
2/3-Phospho-D-glycerate
1-3-Bisphosphoglycerate
Phosphoenolpyruvate
Carnosine
3-Oxalomalate
(5-L-Glutamyl)-L-glutamine
N-Succinyl-L-citrulline
Dopamine
4-Aminobenzoate
Hexanoic acid (caproate)
3-Phosphonopyruvate
Sphingosine 1-phosphate
Sphingosine 1 phosphate (S1P)
D-Arabitol
2-Oxoglutarate
D-glucono-1,5-lactone
3D-(3-5/4)-Trihydroxycyclohexane-1-2-dione
12-HEPE
α-Linoleic acid
Linoleate
Octadecenoic acid (Oleic acid)
13(S)-HODE
12(13)-EpOME
13-OxoODE
9-oxoODE
5(S)-HETE
LPA 14:0
14-HDoHE
8-HETE
11-HETE
(5Z-8Z-11Z-14Z-17Z)-Icosapentaenoic acid
Icosapentaenoic acid

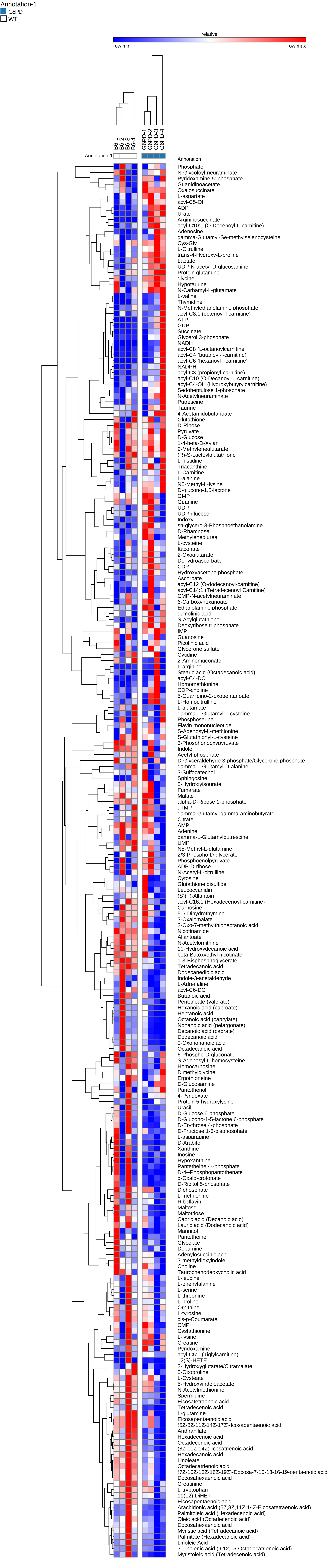
Annotation-1
G6PD
WT
relative
row min
row max
B6-1
B6-2
B6-3
B6-4
G6PD-1
G6PD-2
G6PD-3
G6PD-4
Annotation-1
Annotation
Phosphate
N-Glycolovl-neuraminate
Pyridoxamine 5'-phosphate
Guanidinoacetate
Oxalosuccinate
L-aspartate
acyl-C5-OH
ADP
Urate
Argininosuccinate
acyl-C10:1 (O-Decenoyl-L-carnitine)
Adenosine
gamma-Glutamyl-Se-methylselenocysteine
Cys-Gly
L-Citrulline
trans-4-Hydroxy-L-proline
Lactate
UDP-N-acetyl-D-glucosamine
Protein glutamine
glycine
Hypotaurine
N-Carbamyl-L-glutamate
L-valine
Thymidine
N-Methylethanolamine phosphate
acyl-C8:1 (octenoyl-l-carnitine)
ATP
GDP
Succinate
Glycerol 3-phosphate
NADH
acyl-C8 (L-octanoylcarnitine)
acyl-C4 (butanoyl-l-carnitine)
acyl-C6 (hexanoyl-l-carnitine)
NADPH
acyl-C3 (propionyl-carnitine)
acyl-C10 (O-Decanoyl-L-carnitine)
acyl-C4-OH (Hydroxybutyrylcarnitine)
Sedoheptulose 1-phosphate
N-Acetylneuraminate
Putrescine
Taurine
4-Acetamidobutanoate
Glutathione
D-Ribose
Pyruvate
D-Glucose
1-4-beta-D-Xylan
2-Methyleneglutarate
(R)-S-Lactoylglutathione
L-histidine
Triacanthine
L-Carnitine
L-alanine
N6-Methyl-L-lysine
D-glucono-1,5-lactone
GMP
Guanine
UDP
UDP-glucose
Indoxyl
sn-glycero-3-Phosphoethanolamine
D-Rhamnose
Methylenediurea
L-cysteine
Itaconate
2-Oxoglutarate
Dehydroascorbate
CDP
Hydroxyacetone phosphate
Ascorbate
acyl-C12 (O-dodecanoyl-carnitine)
acyl-C14:1 (Tetradecenoyl Carnitine)
CMP-N-acetylneuraminate
6-Carboxyhexanoate
Ethanolamine phosphate
quinolinic acid
S-Acylglutathione
Deoxyribose triphosphate
IMP
Guanosine
Picolinic acid
Glycerone sulfate
Cytidine
2-Aminomuconate
L-arginine
Stearic acid (Octadecanoic acid)
acyl-C4-DC
Homomethionine
CDP-choline
5-Guanidino-2-oxopentanoate
L-Homocitrulline
L-glutamate
gamma-L-Glutamyl-L-cysteine
Phosphoserine
Flavin mononucleotide
S-Adenosyl-L-methionine
S-Glutathionyl-L-cysteine
3-Phosphonooxypyruvate
Indole
Acetyl phosphate
D-Glyceraldehyde 3-phosphate/Glycerone phosphate
gamma-L-Glutamyl-D-alanine
3-Sulfocatechol
Sphingosine
5-Hydroxyisourate
Fumarate
Malate
alpha-D-Ribose 1-phosphate
dTMP
gamma-Glutamyl-gamma-aminobutyrate
Citrate
AMP
Adenine
gamma-L-Glutamylputrescine
UMP
N5-Methyl-L-glutamine
2/3-Phospho-D-glycerate
Phosphoenolpyruvate
ADP-D-ribose
N-Acetyl-L-citrulline
Cytosine
Glutathione disulfide
Leucocyanidin
(S)(+)-Allantoin
acyl-C16:1 (Hexadecenoyl-carnitine)
Carnosine
5-6-Dihydrothymine
3-Oxalomalate
2-Oxo-7-methylthioheptanoic acid
Nicotinamide
Allantoate
N-Acetylornithine
10-Hydroxydecanoic acid
beta-Butoxyethyl nicotinate
1-3-Bisphosphoglycerate
Tetradecanoic acid
Dodecanedioic acid
Indole-3-acetaldehyde
L-Adrenaline
acyl-C6-DC
Butanoic acid
Pentanoate (valerate)
Hexanoic acid (caproate)
Heptanoic acid
Octanoic acid (caprylate)
Nonanoic acid (pelargonate)
Decanoic acid (caprate)
Dodecanoic acid
9-Oxononanoic acid
Octadecanoic acid
6-Phospho-D-gluconate
S-Adenosyl-L-homocysteine
Homocarnosine
Dimethylglycine
Ergothioneine
D-Glucosamine
Pantothenol
4-Pyridoxate
Protein 5-hydroxylysine
Uracil
D-Glucose 6-phosphate
D-Glucono-1-5-lactone 6-phosphate
D-Erythrose 4-phosphate
D-Fructose 1-6-bisphosphate
L-asparagine
D-Arabitol
Xanthine
Inosine
Hypoxanthine
Pantetheine 4--phosphate
D-4--Phosphopantothenate
α-Oxalo-crotonate
D-Ribitol 5-phosphate
Diphosphate
L-methionine
Riboflavin
Maltose
Maltotriose
Capric acid (Decanoic acid)
Lauric acid (Dodecanoic acid)
Mannitol
Pantetheine
Glycolate
Dopamine
Adenylosuccinic acid
3-methyldioxyindole
Choline
Taurochenodeoxycholic acid
L-leucine
L-phenylalanine
L-serine
L-threonine
L-proline
Ornithine
L-tyrosine
cis-p-Coumarate
CMP
Cystathionine
L-lysine
Creatine
Pyridoxamine
acyl-C5:1 (Tiglylcarnitine)
12(S)-HETE
2-Hydroxyglutarate/Citramalate
5-Oxoproline
L-Cysteate
5-Hydroxyindoleacetate
N-Acetylmethionine
Spermidine
Eicosatetraenoic acid
Tetradecenoic acid
L-glutamine
Eicosapentaenoic acid
(5Z-8Z-11Z-14Z-17Z)-Icosapentaenoic acid
Anthranilate
Hexadecenoic acid
Octadecenoic acid
(8Z-11Z-14Z)-Icosatrienoic acid
Hexadecanoic acid
Linoleate
Octadecatrienoic acid
(7Z-10Z-13Z-16Z-19Z)-Docosa-7-10-13-16-19-pentaenoic acid
Docosahexaenoic acid
Creatinine
L-tryptophan
11(12)-DiHET
Eicosapentaenoic acid
Arachidonic acid (5Z,8Z,11Z,14Z-Eicosatetraenoic acid)
Palmitoleic acid (Hexadecenoic acid)
Oleic acid (Octadecenoic acid)
Docosahexaenoic acid
Myristic acid (Tetradecanoic acid)
Palmitate (Hexadecanoic acid)
Linoleic Acid
?-Linolenic acid (9,12,15-Octadecatrienoic acid)
Myristoleic acid (Tetradecenoic acid)

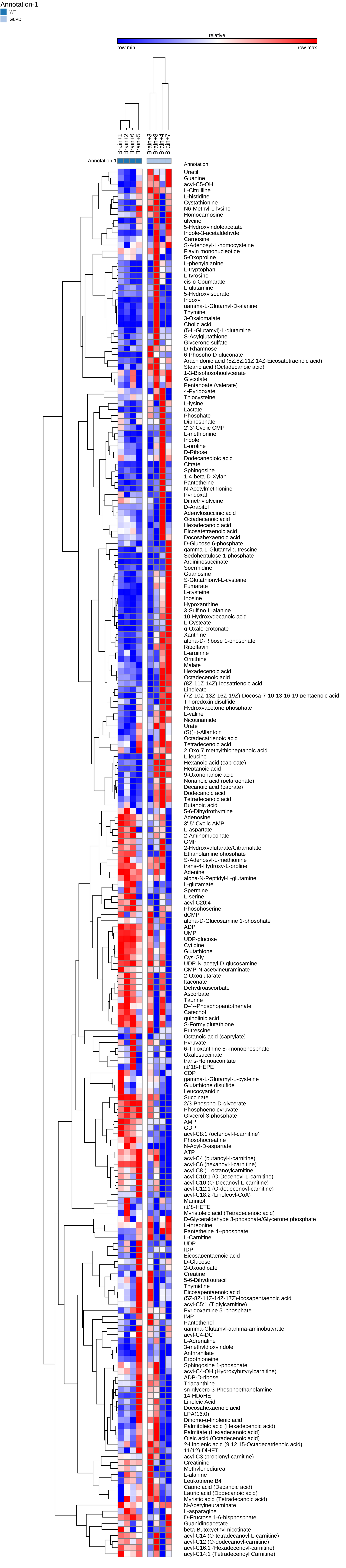
Annotation-1
WT
G6PD
relative
row min
row max
Brain+1
Brain+2
Brain+6
Brain+5
Brain+3
Brain+8
Brain+4
Brain+7
Annotation-1
Annotation
Uracil
Guanine
acyl-C5-OH
L-Citrulline
L-histidine
Cystathionine
N6-Methyl-L-lysine
Homocarnosine
glycine
5-Hydroxyindoleacetate
Indole-3-acetaldehyde
Carnosine
S-Adenosyl-L-homocysteine
Flavin mononucleotide
5-Oxoproline
L-phenylalanine
L-tryptophan
L-tyrosine
cis-p-Coumarate
L-glutamine
5-Hydroxyisourate
Indoxyl
gamma-L-Glutamyl-D-alanine
Thymine
3-Oxalomalate
Cholic acid
(5-L-Glutamyl)-L-glutamine
S-Acylglutathione
Glycerone sulfate
D-Rhamnose
6-Phospho-D-gluconate
Arachidonic acid (5Z,8Z,11Z,14Z-Eicosatetraenoic acid)
Stearic acid (Octadecanoic acid)
1-3-Bisphosphoglycerate
Glycolate
Pentanoate (valerate)
4-Pyridoxate
Thiocysteine
L-lysine
Lactate
Phosphate
Diphosphate
2',3'-Cyclic CMP
L-methionine
Indole
L-proline
D-Ribose
Dodecanedioic acid
Citrate
Sphingosine
1-4-beta-D-Xylan
Pantetheine
N-Acetylmethionine
Pyridoxal
Dimethylglycine
D-Arabitol
Adenylosuccinic acid
Octadecanoic acid
Hexadecanoic acid
Eicosatetraenoic acid
Docosahexaenoic acid
D-Glucose 6-phosphate
gamma-L-Glutamylputrescine
Sedoheptulose 1-phosphate
Argininosuccinate
Spermidine
Guanosine
S-Glutathionyl-L-cysteine
Fumarate
L-cysteine
Inosine
Hypoxanthine
3-Sulfino-L-alanine
10-Hydroxydecanoic acid
L-Cysteate
α-Oxalo-crotonate
Xanthine
alpha-D-Ribose 1-phosphate
Riboflavin
L-arginine
Ornithine
Malate
Hexadecenoic acid
Octadecenoic acid
(8Z-11Z-14Z)-Icosatrienoic acid
Linoleate
(7Z-10Z-13Z-16Z-19Z)-Docosa-7-10-13-16-19-pentaenoic acid
Thioredoxin disulfide
Hydroxyacetone phosphate
L-valine
Nicotinamide
Urate
(S)(+)-Allantoin
Octadecatrienoic acid
Tetradecenoic acid
2-Oxo-7-methylthioheptanoic acid
L-leucine
Hexanoic acid (caproate)
Heptanoic acid
9-Oxononanoic acid
Nonanoic acid (pelargonate)
Decanoic acid (caprate)
Dodecanoic acid
Tetradecanoic acid
Butanoic acid
5-6-Dihydrothymine
Adenosine
3',5'-Cyclic AMP
L-aspartate
2-Aminomuconate
GMP
2-Hydroxyglutarate/Citramalate
Ethanolamine phosphate
S-Adenosyl-L-methionine
trans-4-Hydroxy-L-proline
Adenine
alpha-N-Peptidyl-L-glutamine
L-glutamate
Spermine
L-serine
acyl-C20:4
Phosphoserine
dCMP
alpha-D-Glucosamine 1-phosphate
ADP
UMP
UDP-glucose
Cytidine
Glutathione
Cys-Gly
UDP-N-acetyl-D-glucosamine
CMP-N-acetylneuraminate
2-Oxoglutarate
Itaconate
Dehydroascorbate
Ascorbate
Taurine
D-4--Phosphopantothenate
Catechol
quinolinic acid
S-Formylglutathione
Putrescine
Octanoic acid (caprylate)
Pyruvate
6-Thioxanthine 5--monophosphate
Oxalosuccinate
trans-Homoaconitate
(±)18-HEPE
CDP
gamma-L-Glutamyl-L-cysteine
Glutathione disulfide
Leucocyanidin
Succinate
2/3-Phospho-D-glycerate
Phosphoenolpyruvate
Glycerol 3-phosphate
AMP
GDP
acyl-C8:1 (octenoyl-l-carnitine)
Phosphocreatine
N-Acyl-D-aspartate
ATP
acyl-C4 (butanoyl-l-carnitine)
acyl-C6 (hexanoyl-l-carnitine)
acyl-C8 (L-octanoylcarnitine
acyl-C10:1 (O-Decenoyl-L-carnitine)
acyl-C10 (O-Decanoyl-l-carnitine)
acyl-C12:1 (O-dodecenoyl-carnitine)
acyl-C18:2 (Linoleoyl-CoA)
Mannitol
(±)8-HETE
Myristoleic acid (Tetradecenoic acid)
D-Glyceraldehyde 3-phosphate/Glycerone phosphate
L-threonine
Pantetheine 4--phosphate
L-Carnitine
UDP
IDP
Eicosapentaenoic acid
D-Glucose
2-Oxoadipate
Creatine
5-6-Dihydrouracil
Thymidine
Eicosapentaenoic acid
(5Z-8Z-11Z-14Z-17Z)-Icosapentaenoic acid
acyl-C5:1 (Tiglylcarnitine)
Pyridoxamine 5'-phosphate
IMP
Panthothenol
gamma-Glutamyl-gamma-aminobutyrate
acyl-C4-DC
L-Adrenaline
3-methyldioxyindole
Anthranilate
Ergothioneine
Sphingosine 1-phosphate
acyl-C4-OH (Hydroxybutyrylcarnitine)
ADP-D-ribose
Triacanthine
sn-glycero-3-Phosphoethanolamine
14-HDoHE
Linoleic Acid
Docosahexaenoic acid
LPA(16:0)
Dihomo-g-linolenic acid
Palmitoleic acid (Hexadecenoic acid)
Palmitate (Hexadecanoic acid)
Oleic acid (Octadecenoic acid)
?-Linolenic acid (9,12,15-Octadecatrienoic acid)
11(12)-DiHET
acyl-C3 (propionyl-carnitine)
Creatinine
Methylenediurea
L-alanine
Leukotriene B4
Capric acid (Decanoic acid)
Lauric acid (Dodecanoic acid)
Myristic acid (Tetradecanoic acid)
N-Acetylneuraminate
L-asparagine
D-Fructose 1-6-bisphosphate
Guanidinoacetate
beta-Butoxyethyl nicotinate
acyl-C14 (O-tetradecanoyl-L-carnitine)
acyl-C12 (O-dodecanoyl-carnitine)
acyl-C16:1 (Hexadecenoyl-carnitine)
acyl-C14:1 (Tetradecenoyl Carnitine)

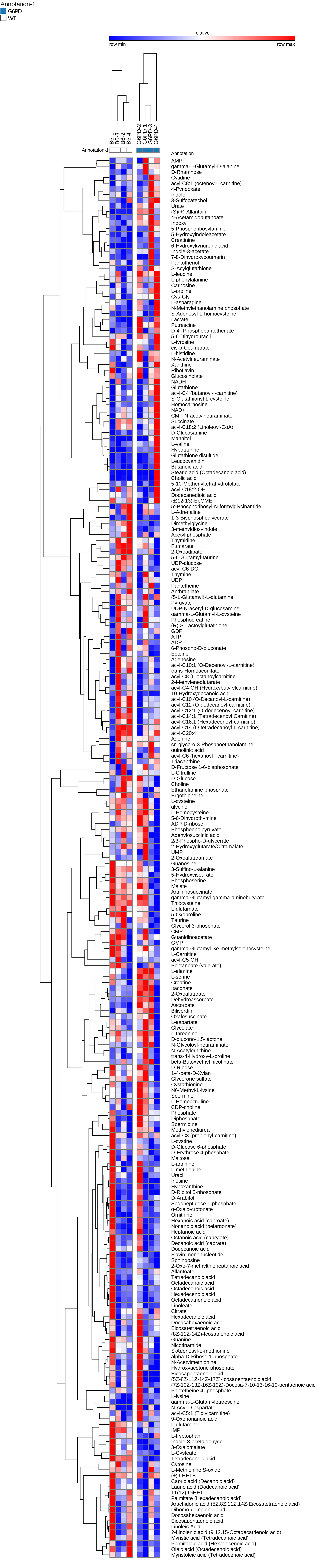

Annotation-1
G6PD
WT
relative
row min
row max
B6-1
B6-3
B6-2
B6-4
G6PD-2
G6PD-1
G6PD-3
G6PD-4
Annotation-1
Annotation
AMP
gamma-L-Glutamyl-D-alanine
D-Rhamnose
Cytidine
acyl-C8:1 (octenoyl-l-carnitine)
4-Pyridoxate
Indole
3-Sulfocatechol
Urate
(S)(+)-Allantoin
4-Acetamidobutanoate
Indoxyl
5-Phosphoribosylamine
5-Hydroxyindoleacetate
Creatinine
6-Hydroxykynurenic acid
Indole-3-acetate
7-8-Dihydroxycoumarin
Pantothenol
S-Acylglutathione
L-leucine
L-phenylalanine
Carnosine
L-proline
Cys-Gly
L-asparagine
N-Methylethanolamine phosphate
S-Adenosyl-L-homocysteine
Lactate
Putrescine
D-4--Phosphopantothenate
5-6-Dihydrouracil
L-tyrosine
cis-p-Coumarate
L-histidine
N-Acetylneuraminate
Xanthine
Riboflavin
Glucosinolate
NADH
Glutathione
acyl-C4 (butanoyl-l-carnitine)
S-Glutathionyl-L-cysteine
Homocarnosine
NAD+
CMP-N-acetylneuraminate
Succinate
acyl-C18:2 (Linoleoyl-CoA)
D-Glucosamine
Mannitol
L-valine
Hypotaurine
Glutathione disulfide
Leucocyanidin
Butanoic acid
Stearic acid (Octadecanoic acid)
Cholic acid
5-10-Methenyltetrahydrofolate
acyl-C18:2-OH
Dodecanedioic acid
(±)12(13)-EpOME
5'-Phosphoribosyl-N-formylglycinamide
L-Adrenaline
1-3-Bisphosphoglycerate
Dimethylglycine
3-methyldioxindole
Acetyl phosphate
Thymidine
Fumarate
2-Oxoadipate
5-L-Glutamyl-taurine
UDP-glucose
acyl-C6-DC
Thymine
UDP
Pantetheine
Anthranilate
(5-L-Glutamyl)-L-glutamine
Pyruvate
UDP-N-acetyl-D-glucosamine
gamma-L-Glutamyl-L-cysteine
Phosphocreatine
(R)-S-Lactoylglutathione
GDP
ATP
ADP
6-Phospho-D-gluconate
Ectoine
Adenosine
acyl-C10:1 (O-Decenoyl-L-carnitine)
trans-Homoaconitate
acyl-C8 (L-octanoylcarnitine
2-Methyleneglutarate
acyl-C4-OH (Hydroxybutyrylcarnitine)
10-Hydroxydecanoic acid
acyl-C10 (O-Decanoyl-L-carnitine)
acyl-C12 (O-dodecanoyl-carnitine)
acyl-C12:1 (O-dodecenoyl-carnitine)
acyl-C14:1 (Tetradecenoyl Carnitine)
acyl-C16:1 (Hexadecenoyl-carnitine)
acyl-C14 (O-tetradecanoyl-L-carnitine)
acyl-C20:4
Adenine
sn-glycero-3-Phosphoethanolamine
quinolinic acid
acyl-C6 (hexanoyl-l-carnitine)
Triacanthine
D-Fructose 1-6-bisphosphate
L-Citrulline
D-Glucose
Choline
Ethanolamine phosphate
Ergothioneine
L-cysteine
glycine
L-Homocysteine
5-6-Dihydrothymine
ADP-D-ribose
Phosphoenolpyruvate
Adenylosuccinic acid
2/3-Phospho-D-glycerate
2-Hydroxyglutarate/Citramalate
UMP
2-Oxoglutaramate
Guanosine
3-Sulfino-L-alanine
5-Hydroxyisourate
Phosphoserine
Malate
Argininosuccinate
gamma-Glutamyl-gamma-aminobutyrate
Thiocysteine
L-glutamate
5-Oxoproline
Taurine
Glycerol 3-phosphate
CMP
Guanidinoacetate
GMP
gamma-Glutamyl-Se-methylselenocysteine
L-Carnitine
acyl-C5-OH
Pentanoate (valerate)
L-alanine
L-serine
Creatine
Itaconate
2-Oxoglutarate
Dehydroascorbate
Ascorbate
Biliverdin
Oxalosuccinate
L-aspartate
Glycolate
L-threonine
D-glucono-1,5-lactone
N-Glycoloyl-neuraminate
N-Acetylornithine
trans-4-Hydroxy-L-proline
beta-Butoxyethyl nicotinate
D-Ribose
1-4-beta-D-Xylan
Glycerone sulfate
Cystathionine
N6-Methyl-L-lysine
Spermine
L-Homocitrulline
CDP-choline
Phosphate
Diphosphate
Spermidine
Methylenediurea
acyl-C3 (propionyl-carnitine)
L-cystine
D-Glucose 6-phosphate
D-Erythrose 4-phosphate
Maltose
L-arginine
L-methionine
Uracil
Inosine
Hypoxanthine
D-Ribitol 5-phosphate
D-Arabitol
Sedoheptulose 1-phosphate
α-Oxalo-crotonate
Ornithine
Hexanoic acid (caproate)
Nonanoic acid (pelargonate)
Heptanoic acid
Octanoic acid (caprylate)
Decanoic acid (caprate)
Dodecanoic acid
Flavin mononucleotide
Sphingosine
2-Oxo-7-methylthioheptanoic acid
Allantoate
Tetradecanoic acid
Octadecanoic acid
Octadecenoic acid
Hexadecenoic acid
Octadecatrienoic acid
Linoleate
Citrate
Hexadecanoic acid
Docosahexaenoic acid
Eicosatetraenoic acid
(8Z-11Z-14Z)-Icosatrienoic acid
Guanine
Nicotinamide
S-Adenosyl-L-methionine
alpha-D-Ribose 1-phosphate
N-Acetylmethionine
Hydroxyacetone phosphate
Eicosapentaenoic acid
(5Z-8Z-11Z-14Z-17Z)-Icosapentaenoic acid
(7Z-10Z-13Z-16Z-19Z)-Docosa-7-10-13-16-19-pentaenoic acid
Pantetheine 4--phosphate
L-lysine
gamma-L-Glutamylputrescine
N-Acyl-D-aspartate
acyl-C5:1 (Tiglylcarnitine)
9-Oxononanoic acid
L-glutamine
IMP
L-tryptophan
Indole-3-acetaldehyde
3-Oxalomalate
L-Cysteate
Tetradecenoic acid
Cytosine
L-Methionine S-oxide
(±)9-HETE
Capric acid (Decanoic acid)
Lauric acid (Dodecanoic acid)
11(12)-DiHET
Palmitate (Hexadecanoic acid)
Arachidonic acid (5Z,8Z,11Z,14Z-Eicosatetraenoic acid)
Dihomo-α-linolenic acid
Docosahexaenoic acid
Eicosapentaenoic acid
Linoleic Acid
?-Linolenic acid (9,12,15-Octadecatrienoic acid)
Myristic acid (Tetradecanoic acid)
Palmitoleic acid (Hexadecenoic acid)
Oleic acid (Octadecenoic acid)
Myristoleic acid (Tetradecenoic acid)

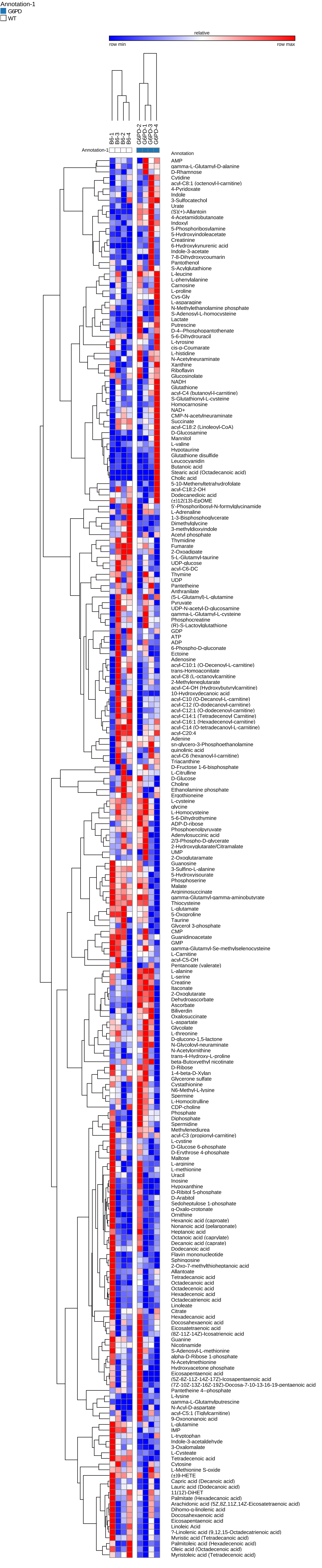

Annotation-1
G6PD
WT
relative
row min
row max
B6-1
B6-3
B6-2
B6-4
G6PD-2
G6PD-1
G6PD-3
G6PD-4
Annotation-1
Annotation
AMP
gamma-L-Glutamyl-D-alanine
D-Rhamnose
Cytidine
acyl-C8:1 (octenoyl-l-carnitine)
4-Pyridoxate
Indole
3-Sulfocatechol
Urate
(S)(+)-Allantoin
4-Acetamidobutanoate
Indoxyl
5-Phosphoribosylamine
5-Hydroxyindoleacetate
Creatinine
6-Hydroxykynurenic acid
Indole-3-acetate
7-8-Dihydroxycoumarin
Pantothenol
S-Acylglutathione
L-leucine
L-phenylalanine
Carnosine
L-proline
Cys-Gly
L-asparagine
N-Methylethanolamine phosphate
S-Adenosyl-L-homocysteine
Lactate
Putrescine
D-4--Phosphopantothenate
5-6-Dihydrouracil
L-tyrosine
cis-p-Coumarate
L-histidine
N-Acetylneuraminate
Xanthine
Riboflavin
Glucosinolate
NADH
Glutathione
acyl-C4 (butanoyl-l-carnitine)
S-Glutathionyl-L-cysteine
Homocarnosine
NAD+
CMP-N-acetylneuraminate
Succinate
acyl-C18:2 (Linoleoyl-CoA)
D-Glucosamine
Mannitol
L-valine
Hypotaurine
Glutathione disulfide
Leucocyanidin
Butanoic acid
Stearic acid (Octadecanoic acid)
Cholic acid
5-10-Methenyltetrahydrofolate
acyl-C18:2-OH
Dodecanedioic acid
(±)12(13)-EpOME
5'-Phosphoribosyl-N-formylglycinamide
L-Adrenaline
1-3-Bisphosphoglycerate
Dimethylglycine
3-methyldioxyindole
Acetyl phosphate
Thymidine
Fumarate
2-Oxoadipate
5-L-Glutamyl-taurine
UDP-glucose
acyl-C6-DC
Thymine
UDP
Pantetheine
Anthranilate
(5-L-Glutamyl)-L-glutamine
Pyruvate
UDP-N-acetyl-D-glucosamine
gamma-L-Glutamyl-L-cysteine
Phosphocreatine
(R)-S-Lactoylglutathione
GDP
ATP
ADP
6-Phospho-D-gluconate
Ectoine
Adenosine
acyl-C10:1 (O-Decenoyl-L-carnitine)
trans-Homoaconitate
acyl-C8 (L-octanoylcarnitine
2-Methyleneglutarate
acyl-C4-OH (Hydroxybutyrylcarnitine)
10-Hydroxydecanoic acid
acyl-C10 (O-Decanoyl-L-carnitine)
acyl-C12 (O-dodecanoyl-carnitine)
acyl-C12:1 (O-dodecenoyl-carnitine)
acyl-C14:1 (Tetradecenoyl Carnitine)
acyl-C16:1 (Hexadecenoyl-carnitine)
acyl-C14 (O-tetradecanoyl-L-carnitine)
acyl-C20:4
Adenine
sn-glycero-3-Phosphoethanolamine
quinolinic acid
acyl-C6 (hexanoyl-l-carnitine)
Triacanthine
D-Fructose 1-6-bisphosphate
L-Citrulline
D-Glucose
Choline
Ethanolamine phosphate
Ergothioneine
L-cysteine
glycine
L-Homocysteine
5-6-Dihydrothymine
ADP-D-ribose
Phosphoenolpyruvate
Adenylosuccinic acid
2/3-Phospho-D-glycerate
2-Hydroxyglutarate/Citramalate
UMP
2-Oxoglutaramate
Guanosine
3-Sulfino-L-alanine
5-Hydroxyisourate
Phosphoserine
Malate
Argininosuccinate
gamma-Glutamyl-gamma-aminobutyrate
Thiocysteine
L-glutamate
5-Oxoproline
Taurine
Glycerol 3-phosphate
CMP
Guanidinoacetate
GMP
gamma-Glutamyl-Se-methylselenocysteine
L-Carnitine
acyl-C5-OH
Pentanoate (valerate)
L-alanine
L-serine
Creatine
Itaconate
2-Oxoglutarate
Dehydroascorbate
Ascorbate
Biliverdin
Oxalosuccinate
L-aspartate
Glycolate
L-threonine
D-glucono-1,5-lactone
N-Glycoloyl-neuraminate
N-Acetylornithine
trans-4-Hydroxy-L-proline
beta-Butoxyethyl nicotinate
D-Ribose
1-4-beta-D-Xylan
Glycerone sulfate
Cystathionine
N6-Methyl-L-lysine
Spermine
L-Homocitrulline
CDP-choline
Phosphate
Diphosphate
Spermidine
Methylenediurea
acyl-C3 (propionyl-carnitine)
L-cystine
D-Glucose 6-phosphate
D-Erythrose 4-phosphate
Maltose
L-arginine
L-methionine
Uracil
Inosine
Hypoxanthine
D-Ribitol 5-phosphate
D-Arabitol
Sedoheptulose 1-phosphate
α-Oxalo-crotonate
Ornithine
Hexanoic acid (caproate)
Nonanoic acid (pelargonate)
Heptanoic acid
Octanoic acid (caprylate)
Decanoic acid (caprate)
Dodecanoic acid
Flavin mononucleotide
Sphingosine
2-Oxo-7-methylthioheptanoic acid
Allantoate
Tetradecanoic acid
Octadecanoic acid
Octadecenoic acid
Hexadecenoic acid
Octadecatrienoic acid
Linoleate
Citrate
Hexadecanoic acid
Docosahexaenoic acid
Eicosatetraenoic acid
(8Z-11Z-14Z)-Icosatrienoic acid
Guanine
Nicotinamide
S-Adenosyl-L-methionine
alpha-D-Ribose 1-phosphate
N-Acetylmethionine
Hydroxyacetone phosphate
Eicosapentaenoic acid
(5Z-8Z-11Z-14Z-17Z)-Icosapentaenoic acid
(7Z-10Z-13Z-16Z-19Z)-Docosa-7-10-13-16-19-pentaenoic acid
Pantetheine 4--phosphate
L-lysine
gamma-L-Glutamylputrescine
N-Acyl-D-aspartate
acyl-C5:1 (Tiglylcarnitine)
9-Oxononanoic acid
L-glutamine
IMP
L-tryptophan
Indole-3-acetaldehyde
3-Oxalomalate
L-Cysteate
Tetradecenoic acid
Cytosine
L-Methionine S-oxide
(±)9-HETE
Capric acid (Decanoic acid)
Lauric acid (Dodecanoic acid)
11(12)-DiHET
Palmitate (Hexadecanoic acid)
Arachidonic acid (5Z,8Z,11Z,14Z-Eicosatetraenoic acid)
Dihomo-α-linolenic acid
Docosahexaenoic acid
Eicosapentaenoic acid
Linoleic Acid
?-Linolenic acid (9,12,15-Octadecatrienoic acid)
Myristic acid (Tetradecanoic acid)
Palmitoleic acid (Hexadecenoic acid)
Oleic acid (Octadecenoic acid)
Myristoleic acid (Tetradecenoic acid)

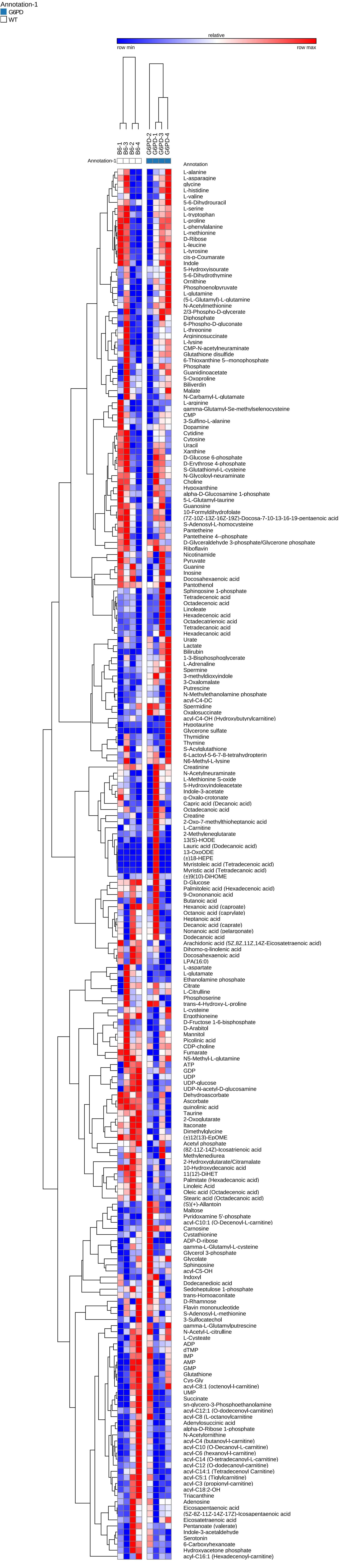

Annotation-1
G6PD
WT
relative
row min
row max
B6-1
B6-3
B6-2
B6-4
G6PD-2
G6PD-1
G6PD-3
G6PD-4
Annotation-1
Annotation
L-alanine
L-asparagine
glycine
L-histidine
L-valine
5-6-Dihydrouracil
L-serine
L-tryptophan
L-proline
L-phenylalanine
L-methionine
D-Ribose
L-leucine
L-tyrosine
cis-p-Coumarate
Indole
5-Hydroxyisourate
5-6-Dihydrothymine
Ornithine
Phosphoenolpyruvate
L-glutamine
(5-L-Glutamyl)-L-glutamine
N-Acetylmethionine
2/3-Phospho-D-glycerate
Diphosphate
6-Phospho-D-gluconate
L-threonine
Argininosuccinate
L-lysine
CMP-N-acetylneuraminate
Glutathione disulfide
6-Thioxanthine 5--monophosphate
Phosphate
Guanidinoacetate
5-Oxoproline
Biliverdin
Malate
N-Carbamyl-L-glutamate
L-arginine
gamma-Glutamyl-Se-methylselenocysteine
CMP
3-Sulfino-L-alanine
Dopamine
Cytidine
Cytosine
Uracil
Xanthine
D-Glucose 6-phosphate
D-Erythrose 4-phosphate
S-Glutathionyl-L-cysteine
N-Glycolyl-neuraminate
Choline
Hypoxanthine
alpha-D-Glucosamine 1-phosphate
5-L-Glutamyl-taurine
Guanosine
10-Formyldihydrofolate
(7Z-10Z-13Z-16Z-19Z)-Docosa-7-10-13-16-19-pentaenoic acid
S-Adenosyl-L-homocysteine
Pantetheine
Pantetheine 4--phosphate
D-Glyceraldehyde 3-phosphate/Glycerone phosphate
Riboflavin
Nicotinamide
Pyruvate
Guanine
Inosine
Docosahexaenoic acid
Pantothenol
Sphingosine 1-phosphate
Tetradecenoic acid
Octadecenoic acid
Linoleate
Hexadecenoic acid
Octadecatrienoic acid
Tetradecanoic acid
Hexadecanoic acid
Urate
Lactate
Bilirubin
1-3-Bisphosphoglycerate
L-Adrenaline
Spermine
3-methyldioxyindole
3-Oxalomalate
Putrescine
N-Methylethanolamine phosphate
acyl-C4-DC
Spermidine
Oxalosuccinate
acyl-C4-OH (Hydroxybutyrylcarnitine)
Hypotaurine
Glycerone sulfate
Thymidine
Thymine
S-Acylglutathione
6-Lactoyl-5-6-7-8-tetrahydropterin
N6-Methyl-L-lysine
Creatinine
N-Acetylneuraminate
L-Methionine S-oxide
5-Hydroxyindoleacetate
Indole-3-acetate
α-Oxalo-crotonate
Capric acid (Decanoic acid)
Octadecanoic acid
Creatine
2-Oxo-7-methylthioheptanoic acid
L-Carnitine
2-Methyleneglutarate
13(S)-HODE
Lauric acid (Dodecanoic acid)
13-OxoODE
(±)18-HEPE
Myristoleic acid (Tetradecenoic acid)
Myristic acid (Tetradecanoic acid)
(±)9(10)-DiHOME
D-Glucose
Palmitoleic acid (Hexadecenoic acid)
9-Oxononanoic acid
Butanoic acid
Hexanoic acid (caproate)
Octanoic acid (caprylate)
Heptanoic acid
Decanoic acid (caprate)
Nonanoic acid (pelargonate)
Dodecanoic acid
Arachidonic acid (5Z,8Z,11Z,14Z-Eicosatetraenoic acid)
Dihomo-α-linolenic acid
Docosahexaenoic acid
LPA(16:0)
L-aspartate
L-glutamate
Ethanolamine phosphate
Citrate
L-Citrulline
Phosphoserine
trans-4-Hydroxy-L-proline
L-cysteine
Ergothioneine
D-Fructose 1-6-bisphosphate
D-Arabitol
Mannitol
Picolinic acid
CDP-choline
Fumarate
N5-Methyl-L-glutamine
ATP
GDP
UDP
UDP-glucose
UDP-N-acetyl-D-glucosamine
Dehydroascorbate
Ascorbate
quinolinic acid
Taurine
2-Oxoglutarate
Itaconate
Dimethylglycine
(±)12(13)-EpOME
Acetyl phosphate
(8Z-11Z-14Z)-Icosatrienoic acid
Methylenediurea
2-Hydroxyglutarate/Citramalate
10-Hydroxydecanoic acid
11(12)-DiHET
Palmitate (Hexadecanoic acid)
Linoleic Acid
Oleic acid (Octadecenoic acid)
Stearic acid (Octadecanoic acid)
(S)(+)-Allantoin
Maltose
Pyridoxamine 5'-phosphate
acyl-C10:1 (O-Decenoyl-L-carnitine)
Carnosine
Cystathionine
ADP-D-ribose
gamma-L-Glutamyl-L-cysteine
Glycerol 3-phosphate
Glycolate
Sphingosine
acyl-C5-OH
Indoxyl
Dodecanedioic acid
Sedoheptulose 1-phosphate
trans-Homoaconitate
D-Rhamnose
Flavin mononucleotide
S-Adenosyl-L-methionine
3-Sulfocatechol
gamma-L-Glutamylputrescine
N-Acetyl-L-citrulline
L-Cysteate
ADP
dTMP
IMP
AMP
GMP
Glutathione
Cys-Gly
acyl-C8:1 (octenoyl-l-carnitine)
UMP
Succinate
sn-glycero-3-Phosphoethanolamine
acyl-C12:1 (O-dodecenoyl-carnitine)
acyl-C8 (L-octanoylcarnitine)
Adenylosuccinic acid
alpha-D-Ribose 1-phosphate
N-Acetylornithine
acyl-C4 (butanoyl-l-carnitine)
acyl-C10 (O-Decanoyl-L-carnitine)
acyl-C6 (hexanoyl-l-carnitine)
acyl-C14 (O-tetradecanoyl-L-carnitine)
acyl-C12 (O-dodecanoyl-carnitine)
acyl-C14:1 (Tetradecenoyl Carnitine)
acyl-C5:1 (Tiglylcarnitine)
acyl-C3 (propionyl-carnitine)
acyl-C18:2-OH
Triacanthine
Adenosine
Eicosapentaenoic acid
(5Z-8Z-11Z-14Z-17Z)-Icosapentaenoic acid
Eicosatetraenoic acid
Pentanoate (valerate)
Indole-3-acetaldehyde
Serotonin
6-Carboxyhexanoate
Hydroxyacetone phosphate
acyl-C16:1 (Hexadecenoyl-carnitine)

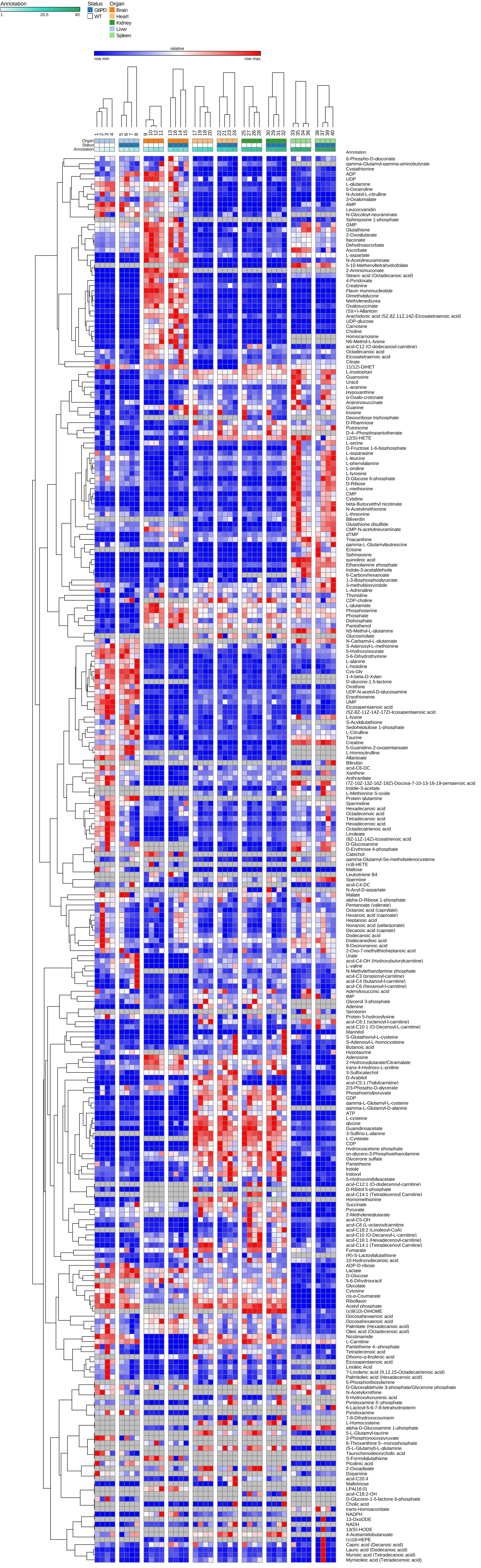
Annotation
1
20.5
40
Status
G6PD
WT
Organ
Brain
Heart
Kidney
Liver
Spleen
relative
row min
row max
Organ
Status
Annotation
Annotation
6-Phospho-D-gluconate
gamma-Glutamyl-gamma-aminobutyrate
Cystathionine
ADP
UDP
L-glutamine
5-Oxoproline
N-Acetyl-L-citrulline
3-Oxalomalate
AMP
Leucocyanidin
N-Glycoloyl-neuraminate
Sphingosine 1-phosphate
GMP
Glutathione
2-Oxoglutarate
Itaconate
Dehydroascorbate
Ascorbate
L-aspartate
N-Acetylneuraminate
5-10-Methenyltetrahydrofolate
2-Aminomuconate
Stearic acid (Octadecanoic acid)
4-Pyridoxate
Creatinine
Flavin mononucleotide
Dimethylglycine
Methylenediurea
Oxalosuccinate
(S)(+)-Allantoin
Arachidonic acid (5Z,8Z,11Z,14Z-Eicosatetraenoic acid)
UDP-glucose
Carnosine
Choline
Homocarnosine
N6-Methyl-L-lysine
acyl-C12 (O-dodecanoyl-carnitine)
Octadecanoic acid
Eicosatetraenoic acid
Citrate
11(12)-DiHET
L-tryptophan
Guanosine
Uracil
L-arginine
Hypoxanthine
α-Oxalo-crotonate
Argininosuccinate
Guanine
Inosine
Deoxyribose triphosphate
D-Rhamnose
Putrescine
D-4--Phosphopantothenate
12(S)-HETE
L-serine
D-Fructose 1-6-bisphosphate
L-asparagine
L-leucine
L-phenylalanine
L-proline
L-tyrosine
D-Glucose 6-phosphate
D-Ribose
L-methionine
CMP
Cytidine
beta-Butoxyethyl nicotinate
N-Acetylmethionine
L-threonine
Biliverdin
Glutathione disulfide
CMP-N-acetylneuraminate
dTMP
Triacanthine
gamma-L-Glutamylputrescine
Ectoine
Sphingosine
quinolinic acid
Ethanolamine phosphate
Indole-3-acetaldehyde
6-Carboxyhexanoate
1-3-Bisphosphoglycerate
3-methyldioxyindole
L-Adrenaline
Thymidine
CDP-choline
L-glutamate
Phosphoserine
Phosphate
Diphosphate
Pantothenol
N5-Methyl-L-glutamine
Glucosinolate
N-Carbamyl-L-glutamate
S-Adenosyl-L-methionine
5-Hydroxyisourate
5-6-Dihydrothymine
L-alanine
L-histidine
Cys-Gly
1-4-beta-D-Xylan
D-glucono-1,5-lactone
Ornithine
UDP-N-acetyl-D-glucosamine
Ergothioneine
UMP
Eicosapentaenoic acid
(5Z-8Z-11Z-14Z-17Z)-Icosapentaenoic acid
L-lysine
S-Acylglutathione
Sedoheptulose 1-phosphate
L-Citrulline
Taurine
Creatine
5-Guanidino-2-oxopentanoate
L-Homocitrulline
Allantoate
Bilirubin
acyl-C6-DC
Xanthine
Anthranilate
(7Z-10Z-13Z-16Z-19Z)-Docosa-7-10-13-16-19-pentaenoic acid
Indole-3-acetate
L-Methionine S-oxide
Protein glutamine
Spermidine
Hexadecanoic acid
Octadecenoic acid
Tetradecanoic acid
Hexadecenoic acid
Octadecatrienoic acid
Linoleate
(8Z-11Z-14Z)-Icosatrienoic acid
D-Glucosamine
D-Erythrose 4-phosphate
Catechol
gamma-Glutamyl-Se-methylselenocysteine
(±)8-HETE
Maltose
Leukotriene B4
Spermine
acyl-C4-DC
N-Acyl-D-aspartate
Malate
alpha-D-Ribose 1-phosphate
Pentanoate (valerate)
Octanoic acid (caprylate)
Hexanoic acid (caproate)
Heptanoic acid
Nonanoic acid (pelargonate)
Decanoic acid (caprate)
Dodecanoic acid
Dodecanedioic acid
9-Oxononanoic acid
2-Oxo-7-methylthioheptanoic acid
Urate
acyl-C4-OH (Hydroxybutyrylcarnitine)
L-valine
N-Methylethanolamine phosphate
acyl-C3 (propionyl-carnitine)
acyl-C4 (butanoyl-l-carnitine)
acyl-C6 (hexanoyl-l-carnitine)
Adenylosuccinic acid
IMP
Glycerol 3-phosphate
Adenine
Serotonin
Protein 5-hydroxylysine
acyl-C8:1 (octenoyl-l-carnitine)
acyl-C10:1 (O-Decenoyl-L-carnitine)
Mannitol
S-Glutathionyl-L-cysteine
S-Adenosyl-L-homocysteine
Butanoic acid
Hypotaurine
Adenosine
2-Hydroxyglutarate/Citramalate
trans-4-Hydroxy-L-proline
3-Sulfocatechol
D-Arabitol
acyl-C5:1 (Tiglylcarnitine)
2/3-Phospho-D-glycerate
Phosphoenolpyruvate
GDP
gamma-L-Glutamyl-L-cysteine
gamma-L-Glutamyl-D-alanine
ATP
L-cysteine
glycine
Guanidinoacetate
3-Sulfino-L-alanine
L-Cysteate
CDP
Hydroxyacetone phosphate
sn-glycero-3-Phosphoethanolamine
Glycerone sulfate
Pantetheine
Indole
Indoxyl
5-Hydroxyindoleacetate
acyl-C12:1 (O-dodecenoyl-carnitine)
D-Ribitol 5-phosphate
acyl-C14:1 (Tetradecenoyl Carnitine)
Homomethionine
Succinate
Pyruvate
2-Methyleneglutarate
acyl-C5-OH
acyl-C8 (L-octanoylcarnitine
acyl-C18:2 (Linoleoyl-CoA)
acyl-C10 (O-Decanoyl-L-carnitine)
acyl-C16:1 (Hexadecenoyl-carnitine)
acyl-C14:1 (Tetradecenoyl Carnitine)
Fumarate
(R)-S-Lactoylglutathione
10-Hydroxydecanoic acid
ADP-D-ribose
Lactate
D-Glucose
5-6-Dihydrouracil
Glycolate
Cytosine
cis-p-Coumarate
Riboflavin
Acetyl phosphate
(±)9(10)-DiHOME
Docosahexaenoic acid
Docosahexaenoic acid
Palmitate (Hexadecanoic acid)
Oleic acid (Octadecenoic acid)
Nicotinamide
L-Carnitine
Pantetheine 4--phosphate
Tetradecenoic acid
Dihomo-g-linolenic acid
Eicosapentaenoic acid
Linoleic Acid
?-Linolenic acid (9,12,15-Octadecatrienoic acid)
Palmitoleic acid (Hexadecenoic acid)
5-Phosphoribosylamine
D-Glyceraldehyde 3-phosphate/Glycerone phosphate
N-Acetylornithine
6-Hydroxykynurenic acid
Pyridoxamine 5'-phosphate
6-Lactoyl-5-6-7-8-tetrahydropterin
Pyridoxamine
7-8-Dihydroxycoumarin
L-Homocysteine
alpha-D-Glucosamine 1-phosphate
5-L-Glutamyl-taurine
3-Phosphonooxypyruvate
6-Thioxanthine 5--monophosphate
(5-L-Glutamyl)-L-glutamine
Taurochenodeoxycholic acid
S-Formylglutathione
Picolinic acid
2-Oxoadipate
Dopamine
acyl-C20:4
Maltotriose
LPA(16:0)
acyl-C18:2-OH
D-Glucono-1-5-lactone 6-phosphate
Cholic acid
trans-Homoaconitate
NADPH
13-OxoODE
NADH
13(S)-HODE
4-Acetamidobutanoate
(±)18-HEPE
Capric acid (Decanoic acid)
Lauric acid (Dodecanoic acid)
Myristic acid (Tetradecanoic acid)
Myristoleic acid (Tetradecenoic acid)
